# Novel Genetic Locus Implicated for HIV-1 Acquisition with Putative Regulatory Links to HIV Replication and Infectivity: A Genome-Wide Association Study

**DOI:** 10.1371/journal.pone.0118149

**Published:** 2015-03-18

**Authors:** Eric O. Johnson, Dana B. Hancock, Nathan C. Gaddis, Joshua L. Levy, Grier Page, Scott P. Novak, Cristie Glasheen, Nancy L. Saccone, John P. Rice, Michael P. Moreau, Kimberly F. Doheny, Jane M. Romm, Andrew I. Brooks, Bradley E. Aouizerat, Laura J. Bierut, Alex H. Kral

**Affiliations:** 1 RTI International, Research Triangle Park, NC, Atlanta, GA, San Francisco, CA, United States of America; 2 Washington University School of Medicine, St. Louis, MO, United States of America; 3 Rutgers University Cell and DNA Repository (RUCDR), Piscataway, NJ, United States of America; 4 Center for Inherited Disease Research (CIDR), Johns Hopkins University, Baltimore, MD, United States of America; 5 School of Nursing, University of California San Francisco, San Francisco, CA, United States of America; 6 Institute for Human Genetics, University of California San Francisco, San Francisco, CA, United States of America; University of Texas Health Science Center San Antonio Texas, UNITED STATES

## Abstract

Fifty percent of variability in HIV-1 susceptibility is attributable to host genetics. Thus identifying genetic associations is essential to understanding pathogenesis of HIV-1 and important for targeting drug development. To date, however, *CCR5* remains the only gene conclusively associated with HIV acquisition. To identify novel host genetic determinants of HIV-1 acquisition, we conducted a genome-wide association study among a high-risk sample of 3,136 injection drug users (IDUs) from the Urban Health Study (UHS). In addition to being IDUs, HIV- controls were frequency-matched to cases on environmental exposures to enhance detection of genetic effects. We tested independent replication in the Women’s Interagency HIV Study (N=2,533). We also examined publicly available gene expression data to link SNPs associated with HIV acquisition to known mechanisms affecting HIV replication/infectivity. Analysis of the UHS nominated eight genetic regions for replication testing. SNP rs4878712 in *FRMPD1* met multiple testing correction for independent replication (P=1.38x10^-4^), although the UHS-WIHS meta-analysis p-value did not reach genome-wide significance (P=4.47x10^-7^ vs. P<5.0x10^-8^) Gene expression analyses provided promising biological support for the protective G allele at rs4878712 lowering risk of HIV: (1) the G allele was associated with reduced expression of *FBXO10* (r=-0.49, P=6.9x10^-5^); (2) *FBXO10* is a component of the Skp1-Cul1-F-box protein E3 ubiquitin ligase complex that targets Bcl-2 protein for degradation; (3) lower *FBXO10* expression was associated with higher *BCL2* expression (r=-0.49, P=8x10^-5^); (4) higher basal levels of Bcl-2 are known to reduce HIV replication and infectivity in human and animal *in vitro* studies. These results suggest new potential biological pathways by which host genetics affect susceptibility to HIV upon exposure for follow-up in subsequent studies.

## Introduction

Susceptibility to acquiring HIV-1 is a heritable trait, with an *in vitro* study estimating that 50% is attributable to host genetics.[1,2] However, HIV infection is a gene-by-environment process requiring exposure. It is likely that multiple HIV exposures are required for infection: 100 incidents of sharing needles with an HIV+ injection drug user (IDU) or 200 incidents of unprotected receptive anal sex with an HIV+ partner being needed, on average, to transmit the virus.[3–5] Thus, accounting for HIV exposure is critical to studying host genetics of HIV acquisition.

Five of seven previous genome-wide association studies (GWAS) of HIV acquisition incorporated measurements of HIV exposure (mother-to-child transmission,[6] serodiscordant heterosextual couples,[7] clinic-based recruitment for sexually transmitted infections (STIs),[8] recruitment of HIV- sex workers,[9] and hemophiliacs with probable exposure[10]), however the studies’ sample sizes were small (n = 226–1,379).[6–10] The two other GWAS of HIV acquisition achieved the largest samples sizes (n = 1,837 and13,851) but used population-based controls who were unlikely to have been exposed to HIV-1.[11,12] None of these prior GWAS identified replicable genes contributing to HIV susceptibility. [1,2,12] Thus, since its discovery in 1996, a 32-base pair deletion in the *CCR5* gene remains the only genetic variant conclusively associated with HIV acquisition.[12–14] Identifying additional genetic associations with HIV acquisition is important to understanding the pathogenesis of HIV-1 and providing targets for medication and vaccine development[1,15] as illustrated by *CCR5Δ32* giving rise to an antiretroviral drug inhibiting viral entry (maraviroc).[13,14]

In this study, we conducted a GWAS of HIV-1 acquisition among a high-risk sample of 3,136 IDUs from the Urban Health Study (UHS). In addition to both cases and controls being IDUs, HIV- controls were frequency-matched to HIV+ cases on a number of exposure risks (e.g., sexual risks)—enhancing detection of genetic contributions to differences in HIV status. We tested for independent replication in the Women’s Interagency HIV Study (WIHS, N = 2,533) and examined gene expression data to link the replicated novel SNP association with HIV acquisition to known mechanisms affecting viral replication and infectivity during acute HIV exposure.

## Materials and Methods

**Fig 1:**
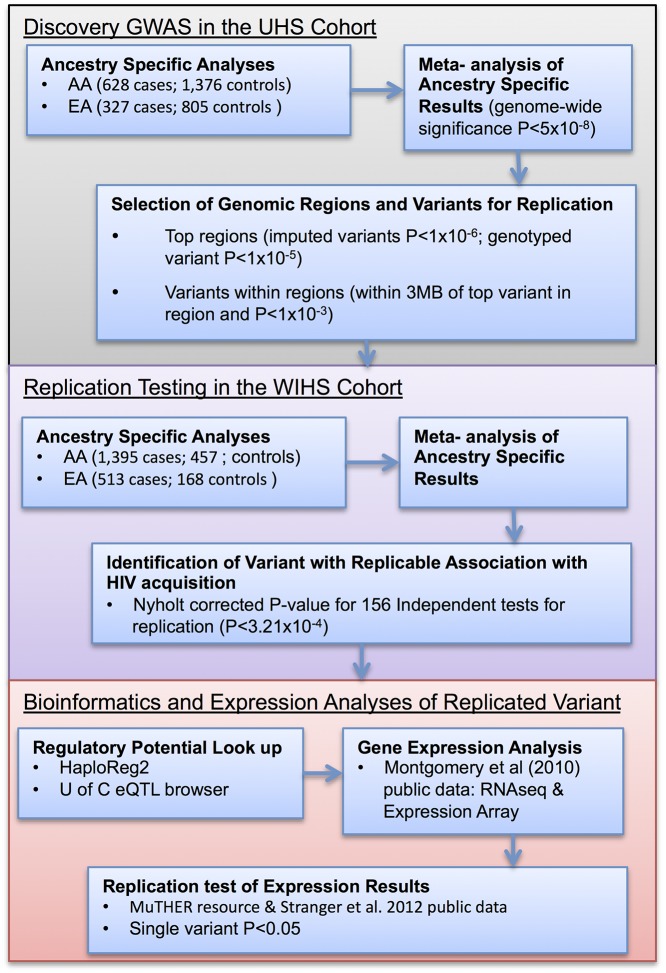
Overview of Study Design. AA represents African Americans, EA represents European Americans, and UofC represents University of Chicago.

In this study we conducted discovery genome-wide association analyses in the UHS cohort, replication testing in the WIHS cohort, and assessment of regulatory potential of replicable variants using publicly available gene expression data. A summary of this study design is presented in Fig. 1, with detailed discussion following.

### Discovery Sample

Study participants were from the UHS, a serial, cross-sectional, sero-epidemiological study of IDUs in the San Francisco Bay Area from 1986 to 2005.[16,17] Study eligibility criteria included injection of an illicit drug in the past 30 days (verified by signs of venipuncture), ability to provide informed consent, age 18 or older, and ability to speak English or Spanish. Participants were interviewed face-to-face regarding key demographics, drug use, and sexual risk behavior. HIV-1 infection status was determined from serum blood samples using enzyme immunoassay and Western Blot assay, identifying HIV+ cases as those who had detectable antibodies.[16,17] The present analysis included self-reported Caucasians (henceforth referred to as European Americans [EAs]) and African Americans (AAs).

### Genome-wide Genotyping and Imputation

All HIV+ cases in the UHS were genotyped. For every case, two HIV- controls were selected for genotyping based on frequency-matching with respect to five criteria: self-identified ancestry, self-identified sex, age group, survey year (pre/post antiretroviral therapy availability), and risk profile that included risky sexual and drug use behaviors (see S1 Methods and S1 Fig.). Genotyping was conducted on 3,732 samples using the Illumina Omni1-Quad BeadChip on restored genomic (not amplified) DNA samples from serum (see S1 Methods). Following quality control (QC), there remained 789,322 autosomal genotyped single nucleotide polymorphisms (SNPs) in 2,017 AAs and 792,340 autosomal genotyped SNPs in 1,142 EAs. Their ancestral proportions are shown in S2 Fig.

Genotype imputation of SNPs and insertion/deletion polymorphisms (indels) was used to expand coverage and increase statistical power.[18] Imputation was conducted in AAs and EAs, separately, using IMPUTE2[18] with reference to the ALL 1000 Genomes reference panel[19] (see S1 Methods).

### Genome-wide Association Analyses

Imputed SNPs and indels were tested for association with HIV-1 case/control status using logistic regression models stratified by ancestry and adjusted for age, sex, behavioral risk class (based on latent class analysis), survey year, and the first 10 principal components to minimize bias due to population stratification (see S1 Methods). The final analysis included 2,004 AAs (628 cases; 1,376 controls) and 1,132 EAs (327 cases; 805 controls) who passed QC and had complete covariate data.

In addition to the ancestral-specific GWAS, we conducted a multi-ancestral meta-analysis to enhance statistical power with a larger sample size. [20,21] The ancestral-specific GWAS results were combined in a fixed-effects sample size-weighted meta-analysis, as done in prior multi-ancestral meta-analyses[22,23], using the METAL program.[24] Meta-analysis results with *P*<5x10^-8^ were considered statistically significant.[25]

### Replication Study Participants and Analyses

Top GWAS meta-analysis results were tested for independent replication in AAs and EAs from the WIHS: the largest longitudinal cohort study of HIV+ and high-risk HIV- women.[26] Similarly to prior GWAS,[27]chromosomal regions from the discovery analysis were selected for replication beyond those with genome-wide significant SNPs. Promising regions / peaks for “deeper” replication testing were selected based imputed SNP/indel associations with P<1x10^-6^ or having the top genotyped SNP association (P = 1x10^-5^), following previously successful studies.[27,28] Each region was defined by 3MB spanning the top associated SNP, given that GWAS signals can reflect synthetic associations as far as 2.5MB away.[29,30] Thus, 692 SNPs and indels with P<1x10^-3^ across the selected regions were tested for replication in WIHS.[28]

All WIHS participants who consented were genotyped on the Illumina Omni2.5 BeadChip using blood as the DNA source. However, only the genotyped SNPs from the 8 selected genomic regions were provided to conduct imputation to the 692 follow-up SNPs and indels that were used for replication testing in the current study. The UHS QC and imputation procedures were repeated for the WIHS participants and their genotyped SNPs from the selected regions. The final analysis data set included 1,852 AAs (1,395 cases; 457 controls) and 681 EAs (513 cases; 168 controls). Imputed SNPs and indels were tested for association with HIV-1 acquisition in logistic regression models adjusted for age, sexual identity (heterosexual, bisexual, lesbian/gay, other), ever use of injected and non-injected drugs, ever had sex with HIV+ male, number of lifetime sexually transmitted diseases (other than HIV and chlamydia), ever had chlamydia, number of sex partners, collection site, wave of recruitment, and 10 principal components. The *P* value threshold for statistically significant replication was 3.21x10^-4^, corresponding to correction for 156 independent tests across the 692 selected SNPs and indels from 8 top gene regions (see S1 Methods).[31,32]

In sum, genome-wide significance threshold was set at P< 5x10^-8^ in the UHS cohort. Given prior successful identification of replicable SNP—disease associations from among signals that were not genome-wide significant in discovery, lower thresholds were used to select regions for follow-up in the WIHS (imputed variants with P<1x10^-6^ and the top genotyped SNP association with P = 1x10^-5^). Within the follow-up regions, variants that had a discovery P<1x10^-3^ within 3MB of the top variant were selected, a total of 692. Taking into account linkage disequilibrium among the 692 follow-up SNPs this constituted 156 independent tests for replication (P<3.21x10^-4^).

### Bioinformatic and Expression Analyses

We evaluated the regulatory potential of replicated findings using the HaploReg v2 database,[33] the University of Chicago expression quantitative trait loci (eQTL) browser, and publically available Montgomery et al.[34] expression array and RNA sequencing data (see S1 Methods). We assessed replication of gene expression findings using Genevar [35]and publically available expression array data from the MuTHER resource[36] and Stranger et al.[37] (see S1 Methods).

### Ethics Statement

The Institutional Review Boards at RTI International and the University of California, San Francisco approved all study procedures for the UHS. The Institutional Review Board at the University of California, San Francisco approved all study procedures for WIHS. All participants in both studies provided written informed consent.

## Results

### GWAS and Replication Cohorts

GWAS and replication testing were conducted using the UHS cohort of high-risk IDUs and the WIHS cohort of high-risk women, respectively (Table 1). By design (S1 Methods), UHS HIV+ cases and HIV- controls have parallel profiles of HIV exposure risk behaviors that enhance detection of genetic associations with HIV acquisition (S1 Table). Although we did not purposefully match HIV+ cases to HIV- controls in the WIHS, WIHS controls are very similar to cases on most HIV exposure risk behaviors and at much higher risk than the general U.S. population due to matched venue/community-based recruitment [26] (S1 Table).

**Table 1:** Characteristics of participants in the Urban Health Study and the Women’s Interagency HIV Study.

**Urban Health Study—Discovery Cohort**			**Women’s Interagency HIV Study—Replication Cohort**		
*Characteristic*	*N = 3*,*136*	*%*	*Characteristic*	*N = 2*,*533*	*%*
HIV Status			HIV Status		
Negative	955	30.4	Negative	1,908	75.3
Positive	2,181	69.6	Positive	625	24.7
Sex			Sex		
Male	781	24.9	Male	2,533	100.0
Female	2,355	75.1	Female	0	0.0
Ancestry			Ancestry		
European American	2,004	63.9	African American	1,852	73.1
African American	1,132	36.1	European American	681	26.9
Year Participated			Recruitment Wave		
1986–1994	1,763	56.2	1994–1995	1,755	69.3
1995–2002	1,373	43.8	2001–2002	778	30.7

### Discovery GWAS

**Fig 2:**
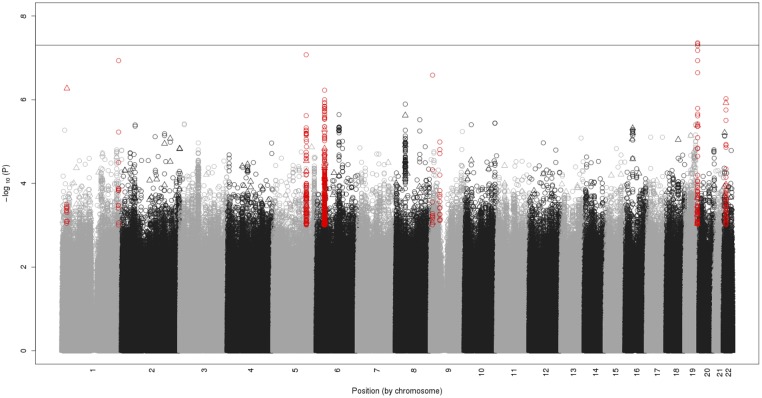
Manhattan plot showing the meta-analysis results of approximately 8 million SNPs and indels tested for association with HIV-1 acquisition in 2,004 African Americans and 1,132 European Americans from the Urban Health Study. The–log_10_ (*P* value) is plotted by chromosomal position of SNPs (shown as circles) and indels (shown as triangles). The SNPs and indels selected for replication testing from 8 gene regions are highlighted in red. The gene region above the solid grey line (*P*<5x10^-8^) exceeded the threshold for genome-wide statistical significance. In addition, the 6 gene regions above the dashed black line (*P*<1x10^-6^) and the region around the top genotyped SNP (*P* = 1x10^-5^ on chromosome 9) were selected for replication testing.

The ancestry-specific GWAS analyses revealed no genome-wide significant associations (P<5x10^-8^, S3 and S4 Figs.). To identify SNP/indel associations with HIV acquisition that are shared across the ancestral groups, we conducted a GWAS meta-analysis of AA and EA IDUs in the UHS cohort based on 8 million imputed SNP and indel genotypes (MAF > 0.5%). The resulting quantile-quantile plot showed some deviation from expectation among top SNP/indel associations but no genomic inflation (λ_gc_ = 1.008; S5 Fig.). We identified one genome-wide significant association on chromosome 19 upstream of the *CD33* gene (rs3987765 meta-analysis p = 4.38x10^-8^) and 6 other regions of interest (P<1x10^-6^). An eighth region on chromosome 9 had the top genotyped SNP association (P = 1.02x10^-5^). The 692 SNPs and indels selected for replication testing from the 8 regions are highlighted in Fig. 2. Their regional association plots from the GWAS meta-analysis are shown in S6 and S7 Figs.

In addition to the top 8 gene regions, we used the UHS meta-analysis results to look-up 24 candidate SNPs that were previously implicated for their suggestive association with HIV-1 acquisition as reviewed by An and Winkler [38] or McLaren et al. [6–9,11,12](S2 Table). None of these previously suggested candidate SNPs had meta-analysis *P*<0.05 in this study.

### Replication Tests in WIHS

The top replication SNP from each of the follow-up regions is presented in Table 2. Results for all tested SNPs and indels are presented in S3 Table. An intronic SNP, rs4878712, in the FERM And PDZ Domain Containing 1 (*FRMPD1*) gene on chromosome 9 replicated at P = 1.38x10^-4^, which surpassed our threshold for multiple testing correction. Its meta-analysis P-value across UHS and WIHS was P = 4.47x10^-7^ with the G allele consistently showing a protective effect for HIV acquisition. The G allele had a lower frequency in cases vs. controls for both ancestry groups in UHS and WIHS: 0.27 vs. 0.33 in UHS AAs, 0.54 vs. 0.56 in UHS EAs, 0.30 vs. 0.35 in WIHS AAs, and 0.52 vs. 0.57 in WIHS EAs. The rs4878712 SNP is located approximately 600kb away from the SNP with the smallest meta-analysis P-value from the discovery analysis of the UHS, rs1329568 (S6H and S7H Figs.). D’ values between rs4878712 and rs1329568 are high in EUR (1.0), but of modest statistical significance, and limited in AFR (0.22) (S8A and S9A Figs.). Their r^2^ values that suggest no correlation are likely constrained by dissimilar allele frequencies [39] (S8B and S9B Figs.). However, examining the haplotypes of the top discovery and top replication SNPs shows the strongest protective effect in the GG haplotype relative to the high risk AT haplotype, with a meta-analysis P-value (P = 5.44x10^-8^) nearly an order of magnitude smaller than the meta-analysis of rs4878712 alone. These results suggest that these SNPs may be tapping into a shared haplotype with a causal variant, representing the same signal. See S4 Table for rs4878712-rs1329568 haplotype analyses by cohort, ancestry, and overall.

**Table 2:** Replication meta-analysis results of SNP associations with HIV acquisition in African Americans and European Americans from the Women’s Interagency HIV Study. Results are presented for the SNP/indel with the best evidence for replication in each GWAS-implicated chromosomal region. These SNPs/indels were selected for replication testing based on having GWAS meta-analysis *P*<1x10^-3^ in each implicated region. SNPs/indels are sorted by their WIHS metaanalysis *P* value. Statistically significant replication was declared where WIHS meta-analysis *P*<3.21x10^-4^ based on correction for multiple testing (shown in bold).

*Chr*: *SNP (coded allele)*	*Position (NCBI build 37)*	*SNP Type*	*Gene / Nearby genes*	*UHS—discovery*							*WIHS—replication*							*UHS and WIHS meta-analysis P*
				*AAs* (N = 2,004)			*EAs* (N = 1,132)			*UHS meta-analysis P*	*AAs* (N = 1,852)			*EAs* (N = 681)			*WIHS meta-analysis P*	
				*CAF*	*P*	*OR (95% CI)*	*CAF*	*P*	*OR (95% CI)*		*CAF*	*P*	*OR (95% CI)*	*CAF*	*P*	*OR (95% CI)*		
9p13.2: rs4878712 (G^b^) **	37,654,257	Intronic	*FRMPD1*	0.31	3.62x10^-4^	0.76 (0.65–0.88)	0.56	0.40	0.92 (0.76–1.12)	7.78x10^-4^	0.31	4.14x10^-4^	0.72 (0.61–0.87)	0.54	0.13	0.80 (0.59–1.07)	**1.38x10** ^**–4**^	4.47x10^-7^
9p24.1: rs16925298 (G) ***	7,081,674	Intronic	*KDM4C*	0.06	0.076	1.30 (0.97–1.72)	0.02	8.03x10^-4^	2.63 (1.49–4.76)	5.96x10^-4^	0.06	4.60x10^-4^	2.00 (1.37–3.03)	0.04	0.63	1.22 (0.55–2.70)	1.18x10^-3^	2.34x10^-6^
6p21.32: rs9272490 (A)	32,606,042	Intronic	*HLA-DQA1*	0.28	0.043	0.85 (0.72–0.99)	0.24	3.12x10^-3^	0.70 (0.55–0.89)	6.94x10^-4^	0.15	2.91x10^-3^	1.47 (1.15–1.92)	0.15	0.30	1.25 (0.81–1.92)	2.08x10^-3^	0.64
5q31.2: rs13154187 (C)	137,768,385	Intronic	*KDM3B*	0.05	7.47x10^-3^	1.51 (1.12–2.05)	0.21	0.023	1.30 (1.04–1.61)	4.51x10^-4^	0.06	3.39x10^-3^	1.89 (1.23–2.86)	0.24	0.44	1.15 (0.81–1.61)	3.64x10^-3^	5.29x10^-6^
19q13.33: rs112231249 (G)	50,713,024	Intronic	*MYH14*	0.13	4.62x10^-3^	1.39 (1.10–1.72)	0.03	5.99x10^-3^	2.13 (1.25–3.70)	9.03x10^-5^	0.13	0.33	1.15 (0.87–1.49)	0.03	0.043	2.70 (1.03–7.14)	0.059	3.01x10^-5^
1q42.3: rs10910535 (T) **	235,096,551	Intergenic	*IRF2BP2 / TOMM20*	0.14	0.15	1.18 (0.94–1.45)	0.28	2.86x10^-4^	1.52 (1.21–1.89)	8.79x10^-4^	0.13	0.49	0.92 (0.71–1.18)	0.26	0.042	0.71 (0.52–0.99)	0.10	0.17
22q12.1: rs137181 (G^a^) ***	26,666,246	Intronic	*SEZ6L*	0.39	2.14x10^-3^	1.24 (1.08–1.43)	0.50	0.086	1.18 (0.98–1.42)	4.91x10^-4^	0.40	0.18	1.12 (0.95–1.34)	0.51	0.37	1.14 (0.85–1.54)	0.11	2.52x10^-4^
1p36.13: chr1: 19357344:D (A)	19,357,344	Intergenic	*IFFO2 / UBR4*	0.18	2.50x10^-4^	0.69 (0.57–0.85)	0.34	5.20x10^-4^	0.69 (0.56–0.85)	5.37x10^-7^	0.18	0.46	1.09 (0.86–1.37)	0.38	0.094	1.30 (0.95–1.75)	0.13	6.47x10^-3^

CI, confidence interval; CAF, coded allele frequency; OR, odds ratio

^a^G is the minor allele for rs137181 in UHS and WIHS AAs, the equi-frequent allele in UHS EAs, and the major allele for WIHS EAs.

^b^G is the minor allele for rs4878712 in UHS and WIHS AAs but the major allele in UHS and WIHS EAs.

Asterisks indicate that the SNP was genotyped in WIHS only (**) or in both UHS and WIHS (***). Otherwise, SNPs were imputed in both study cohorts.

Two additional chromosomal regions harbored SNPs with nominal evidence of replication (P≤3.64x10^-3^): rs13154187 in the Lysine (K)-Specific Demethylase 3B (*KDM3B*) gene on chromosome 5 and rs16925298 in the Lysine (K)-Specific Demethylase 4C (*KDM4C*) gene on chromosome 9. Although Major Histocompatibility Complex, Class II, DQ Alpha 1 (*HLA-*DQA1) SNPs on chromosome 6 also had nominal associations with HIV status in WIHS, opposing directions of association were observed between UHS and WIHS (Table 2). The genome-wide significant finding on chromosome 19 observed in UHS was not replicated in WIHS: rs3987765 replication p = 0.47.

### Bioinformatics and Expression Analyses of *FRMPD1* and HIV-1

We evaluated the *FRMPD1* SNP rs4878712 for its regulatory potential via the University of Chicago eQTL, which identified this SNP as an eQTL for the F-box Protein 10 (*FBXO10*) gene in lymphoblastoid cells lines (LCL). Our further examination of the available Montgomery et al. RNA-sequencing data,[34] showed that the minor G allele, which reduced risk of HIV acquisition, significantly reduced exon 11 expression in *FBXO10* (r = -0.49, P = 6.9 x 10^-5^). No other RNAseq data reporting results for *FBXO10* and rs4878712 in LCL were publically available. Examining publically available micro-array gene expression data, we observed an independent corroborating inverse association between rs4878712 and *FBXO10* in LCL for the gene expression probe ILM_2089616 located in exons 9/10 (β = -0.028, *P* = 0.0176; MuTHER resource[35,36]). However, no association was seen between rs4878712 and the *FBXO10* probe ILM_1716952, which is located farther away in exons 4/5, in two independent datasets (the MuTHER resource P = 0.962; Stranger et al. 2012[37]; *P* = 0.567). The ILM_2089616 probe with suggestive corroborating evidence was not available in the Stranger et al. 2012 data. Finding evidence of reduced expression of *FBXO10* associated with the rs4878712-G allele from two datasets with probes near the 3’ end of the gene but not for a probe toward the 5’ end of the gene may reflect differences in quality of the expression signal from the different probes or the probes tagging different gene transcripts (S10 Fig.).

**Fig 3:**
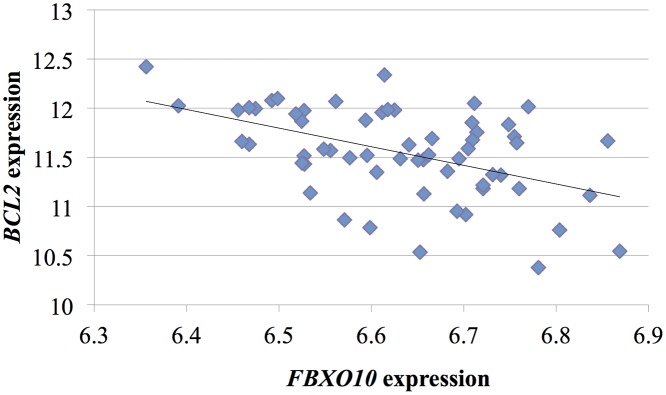
Correlation between *FBXO10* and *BCL2* gene expression. Individual data points for 60 HapMap CEU samples are presented as blue dots, and the linear trend line is shown in black. Microarray data generated and made publically available by Montgomery et al. [34].

The observed reduced expression of *FBXO10* associated with the G allele of rs4878712 may have biological links to risk of HIV acquisition. *FBXO10* is a component of a Skp1-Cul1-F-box protein (SCF) E3 ubiquitin ligase complex that directly targets Bcl-2 protein for degradation.[40] There is interplay between Bcl-2 and HIV in a number of ways over the course of infection,[41] but in the acute phase, higher levels of Bcl-2 are protective *in vitro* and in animal models.[42,43] Thus, lower levels of *FBXO10* expression could be expected to lead to less tagging of Bcl-2 protein for degradation, higher levels of Bcl-2, and greater protection against HIV. Consistent with this possibility, we observed an inverse association between expression of *FBXO10* and *BCL2* (r = -0.49, P = 8 x10^-5^; Fig. 3).

## Discussion

**Fig 4:**
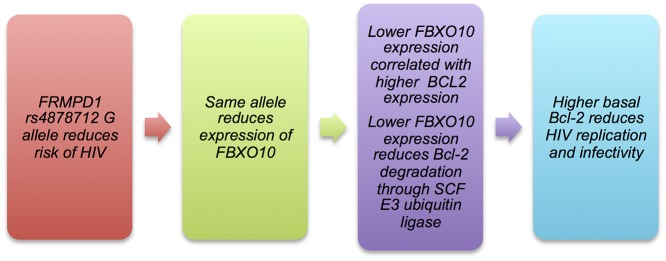
Putative pathway between *FRMPD1* SNP rs4878712 and reduced risk of HIV acquisition through *FBXO10* and *BCL2*/Bcl-2.

This study identified and replicated a promising novel association between rs4878712, located in the *FRMPD1* gene, and HIV acquisition. *FRMPD1* has not been previously associated with HIV and its function is unclear, though it may play a role in subcellular location of activator of G-protein signaling 3 (AGS3)[44] and interact with Leu-Gly-Asn repeat-enriched protein (LGN).[45] Analysis of gene expression data revealed that rs4878712 is an exon-level eQTL for the *FBXO10* gene and that *FBXO10* expression is inversely associated with *BCL2* expression: the HIV-protective G allele reducing *FBXO10* expression, and reduced *FBXO10* expression being associated with increased expression of *BCL2* in healthy lymphoblastoid cells. *FBXO10* is part of an SCF E3 ubiquitin ligase that targets Bcl-2 protein for degradation[40] and higher basal level of Bcl-2 protein is linked to reduced viral replication and infectivity of HIV in the acute phase, potentially distinguishing those who will have an acute infection and those who will develop a persistent one.[42] We hypothesize that Bcl-2 upregulation may be assisted by the putative effect of the rs4878712-G allele on reducing *FBXO10* expression, providing less SCF E3 ubiquitin ligase to tag Bcl-2 for degradation and higher basal *BCL2* expression. Our combination of gene expression evidence and extant literature is consistent with a plausible mechanism linking rs4878712 to acute response to HIV exposure (Fig. 4).

The SNP rs4878712 could be linked with HIV in at least two other ways. First, a recent study of *FBXO10* as a potential oncogene found that manipulation of Lens epithelium-derived growth factor/p75 (LEDGF/p75) protein was positively correlated with *FBXO10* expression in a cellular oxidase stress model. LEDGF/p75 is a key co-factor tethering HIV DNA to host DNA and directing viral DNA integration.[46] Depletion or knockdown of LEDGF/p75 substantially reduces infectivity of the virus.[47] If lower *FBXO10* expression reduces available LEDGF/p75, then it may contribute to protection from HIV infection. Second, ENCODE data identifies rs4878712 as modifying the regulatory motif PRDM1_disc1, suggesting that rs4878712 may alter the transcription binding site for PRDI-BF1 on the *FRMPD1* gene. Of note, the PRDI-BF1 (or BLIMP-1) protein is a transcriptional repressor broadly implicated in T-cell inhibition during HIV infection.[48]

Nominally replicated SNP association signals in the *KDM3B* and *KDM4C* genes are also of potential interest. Both genes function to demethylate Lysine 9 at histone 3 (H3K9).[49] Methylation state of this histone tail site plays a role in silencing/activating HIV transcription at the 5’ end of the long terminal repeats: H3K9 sites are highly methylated in silenced latent HIV, generating a reservoir of virus that is unaffected by the immune system and highly active antiretroviral therapy (HAART).[50] Reactivation of HIV transcription is accompanied by a drop in trimethylation of H3K9,[50] and *KDM4C* is known to convert trimethylated to dimethylated histone residues.[49]

This study’s novel findings may have been enabled by its unique design. Unlike prior GWAS of HIV acquisition, the discovery UHS data set matched HIV- IDU controls to the HIV+ IDU cases on several HIV risk behaviors (see S1 Methods and S1 Fig.), largely equating measurable risk of HIV exposure within this high-risk cohort (S1 Table) and, in theory, improving our statistical power to detect genetic associations with HIV acquisition.

Five prior GWAS of HIV acquisition used other measures of HIV exposure to define HIV- controls including: mother-to-child transmission,[6] recruitment from an STI clinic,[8] recruitment of HIV- sex workers,[9] and hemophiliacs with probable exposure.[10] However, these studies did not further equalize degree of HIV exposure between cases and controls. An exception is Lingappa et al’s study of serodiscordant heterosexual couples,[7] wherein non-seroconverting couples where matched to seroconverting couples on baseline HIV exposure risk based on unprotected sex with HIV+ partner, male uninfected partner uncircumcised, uninfected partner age <25 years, and infected partner plasma viral RNA level. Further, controls for HIV acquisition analyses were selected based on two levels of high HIV exposure scores. The sample sizes for these 5 GWAS were small, ranging from 226 to 1,379 participants. Two other GWAS of HIV acquisition used population controls.[11,12] Although the most recent GWAS used the largest sample size to date (N = 13,851),[12] the vast majority of population controls are unlikely to have been exposed to HIV. Without exposure to the virus, such controls may be minimally informative for studying host genetics of HIV-1 acquisition, suggesting that even larger sample sizes will be required for sufficient statistical power. We assessed top GWAS signals and candidate genes reported in the prior GWAS,[6–12] but did not find any other evidence of replicable association between the previously implicated variants and HIV acquisition in the UHS cohort (P>0.05, see S2 Table). Prior suggestive findings may not be truly associated; we may remain underpowered to adequately test these associations; and/or the difference in types (sexual vs. drug injection) or degree of HIV exposure across studies may limit the field’s ability to replicate findings.

Although this study has several strengths, there are limitations. First, and most notably, the SNPs with the best evidence for replication were not the top SNP associations from the discovery analysis. For replication, we took all SNPs with P<1x10^-3^ that were within 3MB of the top discovery SNP for each signal based on the recognition that variants with the top statistical association signals and the underlying true causal variants may not be the same.[29,30] Although this is a broad replication strategy and the meta-analysis P value does not meet genome-wide significance (P = 4.47x10^-7^ vs. P<5.0x10^-8^), we applied appropriate multiple testing correction and identified a SNP association that surpassed the significance threshold for replication. Haplotype analyses of the top replication SNP (rs4878712) and the discovery SNP on chromosome 9 (rs1329568) suggested a stronger association when considering the paired protective alleles (meta-analysis P = 5.44x10^-8^) than rs4878712 alone (meta-analysis P = 4.47x10^-7^), which may indicate a shared haplotype with a causal variant representing a single signal. Second, although different types of HIV exposure were present in both the discovery and replication cohorts, differences in the predominate modes of HIV exposure between the UHS IDUs and the all female WIHS cohort would tend to emphasize genetic factors that are common across modes of exposure and could have limited our ability to replicate findings. Another limitation is that the gene expression analyses in this study are limited by the publically available data. The Montgomery et al.[34] RNAseq data provided the strongest evidence of rs4878712 as an eQTL for *FBXO10*, particularly for exon 11. The MuTHER resource data[36] provided corroborating evidence of reduced *FBXO10* expression associated with the rs4878712 G allele for an expression array probe located near exon 11. However, a more distal probe near exons 4/5 did not show such an association. Additionally, the available gene expression data are from subjects of European ancestry. Analysis of African American samples in the future would be of significant value. It will also be of value for future studies to move beyond the *in vitro* and animal model studies to test the putative linkage of BCL2/Bcl-2 to HIV infectivity in humans. Nonetheless, the gene expression analyses presented in this study suggest a novel and biologically plausible role for the identified SNP (rs4878712) in HIV acquisition.

In this study we identified and independently replicated a novel association between a variant in the *FRMPD1* gene and HIV acquisition. The magnitude of the replicable association between this newly implicated SNP (rs4878712) and HIV acquisition is modest. Nonetheless, the potential pathway we present (rs4878712 to *FBXO10* and *FBXO10* to *BCL2*/Bcl-2) has good biological plausibility, given the observed protection against viral replication and lower level of infectivity *in vitro* due to basal level of Bcl-2. This or other pathways associated with rs4878712 could be important mechanisms contributing to the variability in susceptibility to HIV infection upon exposure and provide new targets for medication development.

## Supporting Information

S1 Methods Supporting Methods Details. (PDF)

S1 Table Known Behavioral Risk of HIV Exposure among HIV+ cases and HIV- controls. (PDF)

S2 Table Associations of 24 candidate SNPs with HIV-1 acquisition in our meta-analysis of African Americans and European Americans from the Urban Health Study. (PDF)

S3 Table Replication meta-analysis results of all tested SNP associations with HIV acquisition in African Americans and European Americans from the Women’s Interagency HIV Study. Results are presented for all 692 SNPs/indels tested for replication in each GWAS-implicated chromosomal region. SNPs/indels are sorted by gene region. UHS, WIHS replication, overall meta-analyses, and ancestry specific meta-analyses are presented.
(XLSX)

S4 Table Haplotype analysis of top replication and top discovery SNPs (rs4878712-rs1329568) in the chromosome 9 follow-up region: by cohort, by ancestry, and overall meta-analysis results for HIV acquisition in the Urban Health Study and the Women’s Interagency HIV Study. Risk haplotype is used as the reference haplotype to match the protective effect for the tested allele for the replication SNP rs4878712.
(XLSX)

S1 Fig Best Fitting Latent Class Model of HIV Risk behavior among IDUs. (PDF)

S2 Fig STRUCTURE triangle plots showing estimated ancestral proportions of African American and European American participants with reference to HapMap populations. (PDF)

S3 Fig Genome-wide association study of HIV-1 acquisition in 2,004 African Americans from the Urban Health Study. (PDF)

S4 Fig Genome-wide association study of HIV-1 acquisition in 1,142 European Americans from the Urban Health Study. (PDF)

S5 Fig Quantile-quantile plot showing the meta-analysis results of approximately 8 million SNPs and indels tested for association with HIV-1 acquisition in 2,004 African Americans and 1,132 European Americans from the Urban Health Study. (PDF)

S6 Fig Regional association results from the GWAS meta-analysis in the Urban Health Study and their linkage disequilibrium patterns with reference to the 1000 Genomes AFR panel. (PDF)

S7 Fig Regional association results from the GWAS meta-analysis in the Urban Health Study and their linkage disequilibrium patterns with reference to the 1000 Genomes EUR panel. (PDF)

S8 Fig Linkage disequilibrium patterns in the 1000 Genomes AFR reference panel for the GWAS-implicated region spanning from *PAX5* to *FRMPD1* on chromosome 9. (PDF)

S9 Fig Linkage disequilibrium patterns in the 1000 Genomes EUR reference panel for the GWAS-implicated region spanning from *PAX5* to *FRMPD1* on chromosome 9. (PDF)

S10 Fig Location of gene expression probes tested for replication of RNAseq association between rs4878712 and *FBXO10*. (PDF)
