# Correction: Novel Genetic Locus Implicated for HIV-1 Acquisition with Putative Regulatory Links to HIV Replication and Infectivity: A Genome-Wide Association Study

**DOI:** 10.1371/journal.pone.0129671

**Published:** 2015-05-29

**Authors:** Eric O. Johnson, Dana B. Hancock, Nathan C. Gaddis, Joshua L. Levy, Grier Page, Scott P. Novak, Cristie Glasheen, Nancy L. Saccone, John P. Rice, Michael P. Moreau, Kimberly F. Doheny, Jane M. Romm, Andrew I. Brooks, Alex H. Kral

There are errors in Table 1, “Characteristics of participants in the Urban Health Study and the Women’s Interagency HIV Study.” Please see the corrected Table 1 here.

**Table 1:** Characteristics of participants in the Urban Health Study and the Women’s Interagency HIV Study.

Urban Health Study—Discovery Cohort				Women’s Interagency HIV Study—Replication Cohort			
*Characteristic*		*N = 3*,*136*	*%*	*Characteristic*		*N = 2*,*533*	*%*
HIV Status				HIV Status			
	Positive	955	30.4		Positive	1,908	75.3
	Negative	2,181	69.6		Negative	625	24.7
Sex				Sex			
	Female	781	24.9		Female	2,533	100.0
	Male	2,355	75.1		Male	0	0.0
Ancestry				Ancestry			
African American		2,004	63.9	African American		681	26.9
European American		1,132	36.1	European American		1,852	73.1
Year Participated				Recruitment Wave			
1986–1994		1,763	56.2	1994–1995		1,755	69.3
1995–2002		1,373	43.8	2001–2002		778	30.7
