# Supplementary material for: Novel Genetic Locus Implicated for HIV-1 Acquisition with Putative Regulatory Links to HIV Replication and Infectivity: A Genome-Wide Association Study
Source: PLoS One. 2015 Mar 18;10(3):e0118149. doi: 10.1371/journal.pone.0118149 (PMC4364715; doi:10.1371/journal.pone.0118149)
Supplement: S1 Methods — (PDF) [file pone.0118149.s001.pdf]

Supporting Methods

Selection of GWAS Study Participants. All HIV-1 positive cases in the Urban Health Study (UHS) were genotyped. Among the larger set of HIV-1 negative controls, selection for genotyping was based on frequency-matching with respect to five criteria: self-identified ancestry, self-identified sex, age group, survey year (1986-1994, 1995-2002), and risk behavior profile. Participants' risk behavior profiles were determined using a Latent Class Analysis (LCA).[1,2] Having identified the key behavioral risk factors associated with HIV status among UHS injection drug users (IDUs) (receptive anal sex, receptive needle sharing, STIs, sex work, and number of sex partners), we first tested a confirmatory factor model, which indicated that the association with HIV risk was nonlinear and a single risk factor score would be insufficient to match controls to cases. Thus a Latent Class Analysis (LCA) procedure was implemented to evaluate the different patterns of risk behavior. The best model fit suggested three distinct patterns of risk (see S1 Figure): a 3-class model fitting significantly better than 2 classes (Likelihood Ratio Test $p = 0.005$) but 4-class not fitting better than the 3-class model (Likelihood Ratio Test $p = 0.11$). Class 1, consisting of 16% of participants, had a large proportion of people indicating having multiple sex partners, engaging in sex work, and having had an STI. They were also fairly likely to report receptive needle sharing, and had the highest probability of endorsing receptive anal sex. Classes 2 (44%) and 3 (40%) both had low endorsement of multiple sex partners, receptive anal sex, and sex work but differed on probabilities of having an STI and receptive needle sharing. Corresponding to these differences in HIV risk behavior, particularly probability of needle sharing, Classes 1 and 2 were significantly more likely to be HIV+ than Class 3, even among IDUs (OR= 2.04, 95% CI 1.74-2.40 & OR= 1.48, 95% CI 1.29-1.87, respectively).

For matching purposes, participants were assigned to one group each based upon their highest posterior probability of group membership. We then examined non-behavioral risk factors to determine the other criteria to match on. Age, race, survey year, and sex were all significantly associated with HIV status and were included, with LCA group membership to determine a final match code (e.g., a group 3, male, African American, surveyed between 1986 and 1994, and under age 37 would be coded as 32210) by which 2 controls were selected for each case. Proportional case/control matching was maintained across and within genotyping plate assignments.

Genotyping and Quality Control. For genotyping of the UHS samples, DNA was extracted from stored serum samples at the Rutgers Cell and DNA Repository. Sample restoration was conducted using the Illumina Formalin-Fixed Paraffin-Embedded (FFPE) kit to maximize genomic DNA quality. The restored genomic (not amplified) DNA samples were genotyped on the Illumina Omni1-Quad BeadChip at the Center for Inherited Disease Research. Genotype concordance rate among HapMap control samples was 99.7%. Blind duplicate samples had a genotype concordance of 99.9%. UHS study genotypes are deposited in the database of Genotypes and Phenotypes (dbGaP) under accession number phs000454.v1.p1.

Quality control (QC) procedures were implemented using PLINK [3], unless otherwise stated, on 3,732 genotyped samples from HIV-1 cases and controls who had an overall genotyping call rate above 90%. Given the serial nature of our study design, we began with preliminary participant-level QC by identifying unexpected sample duplicates. Using an identity-by-state (IBS) threshold of 90%, we identified 409 unexpected duplicate pairs/clusters. For each duplicate pair or cluster involving HIV-positive cases, we selected the sample with the highest call rate, except in a few instances where we selected an HIV-positive sample over a HIV-

negative sample, which likely occurred if the HIV virus was acquired between survey years. For each duplicate pair or cluster involving only HIV-negative controls, we selected the sample with the highest call rate. One pair of intended blind duplicates was found to come from two distinct individuals, so these two samples were excluded from further analysis.

The STRUCTURE program [4] was used to assess ancestral misclassification via comparison to HapMap phase III reference populations of West Africans (denoted YRI), European Americans (denoted CEU), and Chinese (denoted CHB) using 10,000 SNPs randomly distributed across the genome. The STRUCTURE results corroborated the self-identified ancestral group for most all samples. The ancestral classification of 23 self-identified African Americans (AAs) was re-assigned to the European American (EA) group, because their genetic data indicated low African ancestry (<25%). Similarly, the ancestral classification of 12 self-identified EAs was re-assigned to the AA group, because of high African ancestry (>25%). Further, two self-identified EAs were excluded due to predominant Asian ancestry (>80%) and little European ancestry (8-12%).

All other QC procedures were implemented separately in the ancestral groups using genotyped SNPs with an rs-identifier, call rate>90%, and Hardy-Weinberg P>0.0001 (809,942 SNPs in AAs and 812,895 SNPs in EAs). Among the 79 intended blind duplicate pairs confirmed using IBS>90%, the sample with the highest call rate was retained leaving 3,240 unique samples from 2,088 AAs and 1,152 EAs. The relatedness between each pair of participants was evaluated using identity-by-descent (IBD) estimates in both ancestral groups. In AAs, relatedness was further evaluated using kinship coefficients from the KING program, which was designed to circumvent the inflation of IBD estimates due to population stratification.[5] We identified first-degree relative pairs/clusters, based on IBD>40% for EAs

and IBD>40% and kinship coefficient>0.177 [5] for AAs, and we retained the participant with the highest call rate from each relative pair or cluster. Next, we identified participants with discordance between reported sex and estimated sex based on heterozygosity estimates from chromosome X SNPs (F_{ST}<0.2 being indicative of females and F_{ST}>0.8 being indicative of males[3]).A few participants with sex discordance had duplicate samples from which we were able to resolve the conflicting sex annotation. None of the participants had excessive homozogosity. Following all QC procedures, there remained 789,322 autosomal genotyped SNPs in 2,017 AAs and 792,340 autosomal genotyped SNPs in 1,142 EAs. The ancestral distributions of the participants passing QC are presented in Figure S2A for AAs and Figure S2B for EAs.

To reduce any residual bias in the genetic association analyses due to population stratification in each resulting ancestral group, we subsequently conducted principal component analyses based on genotyped SNPs in linkage equilibrium (pairwise r^2<0.2) using the EIGENSTRAT program.[6] The first 10 principal components were included as covariates in the regression models used to test genetic associations with HIV case/control status.

Genotype Imputation. Among several imputation software programs that are available, we previously found that IMPUTE2 [7] provides the highest imputation quality for AA studies, and we recommended using the 1000 Genomes "cosmopolitan" panel[8] consisting of all available reference subjects from 14 diverse populations (denoted ALL) instead of using smaller panels consisting of more closely related reference subjects [9]. Others have recommended the "cosmopolitan" panel as well[7,10,11]. Our imputation strategy was further optimized by filtering out SNPs with MAF <0.5% within each ancestry group.

Our imputation procedure was conducted separately by ancestral group, using IMPUTE2 version 2.2.2 [7] and reference haplotype panels from 1,092 subjects available in the March 2012 release of 1000 Genomes (http://mathgen.stats.ox.ac.uk/impute/data_download_1000G_phase1_integrated.html, release version 3 downloaded on April 19, 2012). The reference panels included both SNP and insertion/deletion (indel) variants. Imputations were preceded by prephasing the study genotypes with the ShapeIT program [12] to estimate haplotypes, using 500 conditioning states, recommended effective population sizes of 15,000 for AAs and 11,418 for EAs, and default settings for all other program options. The estimated study haplotypes were then used by IMPUTE2 to impute SNP and indel genotypes based on the highly dense set of SNPs and indels on the 1000 Genomes ALL reference haplotype panel. Imputations were conducted on 4.5 MB chunks with 1MB flanking buffers. Default options were used, except for specifying "k_hap" as 468 haplotypes for AAs and 170 haplotypes for EAs. The IMPUTE2 probabilities for each of the three genotypes were converted into a single imputed genotype dosage value (a fractional value between 0 and 2 indicating the expected number of minor allele copies) to use for association testing.

Determining P-value Threshold for Replication. To correct for multiple testing, Bonferroni correction for the total number of SNPs ($P < \alpha/M$, where M is the number of independent tests) is too conservative, given the correlation patterns among SNPs. Therefore, we applied the widely used Nyholt correction ($P < \alpha/M_{eff}$), which adjusts for the effective number of independent tests (M_{eff}) based on the correlation matrix of pairwise linkage disequilibrium (LD) among the SNPs.[13,14] This method gave significance threshold of $P < 3.21 \times 10^{-4}$.

Bioinformatics analyses. We evaluated the regulatory potential of significantly replicated SNP findings using three bioinformatics resources. First, the HaploReg v2 database[15] was used to annotate SNP regulatory potential based on chromatin states, conservation, and regulatory motif alterations identified from the Encyclopedia of DNA Elements (ENCODE), the Roadmap Epigenome Mapping Consortium, the Genotype-Tissue Expression project (GTEx), and elsewhere. Second, the expression quantitative trait loci (eQTL) browser available at http://eqtl.uchicago.edu/cgi-bin/gbrowse/eqtl/ (last accessed September 22, 2013) displays publically available results deposited by several large-scale eQTL studies that collectively assayed multiple tissue and cell types. The Montgomery et al. study[16] was particularly relevant for our study in uncovering significant expression patterns using RNA-sequencing data on lymphoblastoid cells from 60 HapMap CEU individuals. In addition to their RNAsequencing data, Montgomery et al. made their Illumina Human-6 v2 Expression BeadChip data publically available at http://jungle.unige.ch/rnaseq_CEU60/. We downloaded the publically available Montgomery et al.[16] data, assessed eQTL associations, and computed the correlation of transcripts encoded by genes of interest, using the BeadChip data. Third, we tested for replication of eQTL associations observed in the Montgomery et al. using publically available gene expression and SNP data meeting the following criteria: (1) included at least one expression probe in *FBXO10* that passed originating study QC; (2) reported results for variants identified as associated with HIV acquisition and gene expression in Montgomery et al.. No replication RNAseq data were identified but two micro-array gene expression studies were available the MuTHER resource[17] and Stranger et al 2012[18], both of which were available through GENe Expression VARiation (Genevar --

http://www.sanger.ac.uk/resources/software/genevar/)[19]. Lookup analysis used the Genevar tool using default analyses and selecting all SNPs.

REFERENCES

1. Goodman LA (1974) The analysis of systems of qualitative variables when some of the variables are unobservable. Part I - a modified latent structure approach. Am J Sociol 79: 1179-1259.
2. Haberman SJ (1979) Analysis of Qualitative Data, Vol 2, New Developments. New York: Academic Press.
3. Purcell S, Neale B, Todd-Brown K, Thomas L, Ferreira MA, et al. (2007) PLINK: a tool set for whole-genome association and population-based linkage analyses. Am J Hum Genet 81: 559-575.
4. Pritchard JK, Stephens M, Donnelly P (2000) Inference of population structure using multilocus genotype data. Genetics 155: 945-959.
5. Manichaikul A, Mychaleckyj JC, Rich SS, Daly K, Sale M, et al. (2010) Robust relationship inference in genome-wide association studies. Bioinformatics 26: 2867-2873.
6. Price AL, Patterson NJ, Plenge RM, Weinblatt ME, Shadick NA, et al. (2006) Principal components analysis corrects for stratification in genome-wide association studies. Nat Genet 38: 904-909.
7. Howie B, Marchini J, Stephens M (2011) Genotype imputation with thousands of genomes. G3 (Bethesda) 1: 457-470.
8. (2010) A map of human genome variation from population-scale sequencing. Nature 467: 1061-1073.
9. Hancock DB, Levy JL, Gaddis NC, Bierut LJ, Saccone NL, et al. (2012) Assessment of Genotype Imputation Performance using 1000 Genomes in African American Studies. PLoS One 7: e50610.
10. Jostins L, Morley KI, Barrett JC (2011) Imputation of low-frequency variants using the HapMap3 benefits from large, diverse reference sets. Eur J Hum Genet 19: 662-666.
11. Marchini J, Howie B (2010) Genotype imputation for genome-wide association studies. Nat Rev Genet 11: 499-511.
12. Howie B, Fuchsberger C, Stephens M, Marchini J, Abecasis GR (2012) Fast and accurate genotype imputation in genome-wide association studies through pre-phasing. Nat Genet 44: 955-959.
13. Li J, Ji L (2005) Adjusting multiple testing in multilocus analyses using the eigenvalues of a correlation matrix. Heredity (Edinb) 95: 221-227.
14. Nyholt DR (2004) A simple correction for multiple testing for single-nucleotide polymorphisms in linkage disequilibrium with each other. Am J Hum Genet 74: 765-769.
15. Ward LD, Kellis M (2012) HaploReg: a resource for exploring chromatin states, conservation, and regulatory motif alterations within sets of genetically linked variants. Nucleic Acids Res 40: D930-934.
16. Montgomery SB, Sammeth M, Gutierrez-Arcelus M, Lach RP, Ingle C, et al. (2010) Transcriptome genetics using second generation sequencing in a Caucasian population. Nature 464: 773-777.
17. Grundberg E, Small KS, Hedman AK, Nica AC, Buil A, et al. (2012) Mapping cis- and trans-regulatory effects across multiple tissues in twins. Nat Genet 44: 1084-1089.
18. Stranger BE, Montgomery SB, Dimas AS, Parts L, Stegle O, et al. (2012) Patterns of cis regulatory variation in diverse human populations. PLoS Genet 8: e1002639.

19. Yang TP, Beazley C, Montgomery SB, Dimas AS, Gutierrez-Arcelus M, et al. (2010) Genevar: a database and Java application for the analysis and visualization of SNP-gene associations in eQTL studies. Bioinformatics 26: 2474-2476.
20. An P, Winkler CA (2010) Host genes associated with HIV/AIDS: advances in gene discovery. Trends Genet 26: 119-131.
21. McLaren PJ, Coulonges C, Ripke S, van den Berg L, Buchbinder S, et al. (2013) Association Study of Common Genetic Variants and HIV-1 Acquisition in 6,300 Infected Cases and 7,200 Controls. PLoS Pathog 9: e1003515.
