# Supplementary material for: Novel Genetic Locus Implicated for HIV-1 Acquisition with Putative Regulatory Links to HIV Replication and Infectivity: A Genome-Wide Association Study
Source: PLoS One. 2015 Mar 18;10(3):e0118149. doi: 10.1371/journal.pone.0118149 (PMC4364715; doi:10.1371/journal.pone.0118149)
Supplement: S1 Table — (PDF) [file pone.0118149.s002.pdf]

Table S1. Known Behavioral Risk of HIV Exposure among HIV+ cases and HIV- controls

Urban Health Study – Discovery Cohort			***Women's Interagency HIV Study – Replication Cohort***		
*Characteristic**	*HIV+ cases (n = 955) %*	*HIV- controls (n = 2,181) %*	*Characteristic***	*HIV+ cases (n = 1,908) %*	*HIV- controls (n = 625) %*
Injection Drug Use	100.0	100.0	Injection Drug Use	34.8	23.4
Non-injection Drug Use	100.0	100.0	Non-injection Drug Use	75.3	82.2
2 or more sexual partners	19.8	19.0	5 or more sexual partners	61.5	66.7
Had STI other than HIV	66.8	61.5	Had STI other than HIV	73.3	58.6
Engaged in sex work	23.8	25.8	Engaged in sex work	30.2	37.5
Engaged in receptive anal sex	15.3	4.9	NA	NA	NA
Engaged in needle sharing	22.6	24.4	NA	NA	NA
NA	NA	NA	Had sex with a man known to have HIV	42.5	13.6

NA, not available

* Characteristic reported for the past 30 days

** Characteristic reported for lifetime
