# Supplementary material for: Novel Genetic Locus Implicated for HIV-1 Acquisition with Putative Regulatory Links to HIV Replication and Infectivity: A Genome-Wide Association Study
Source: PLoS One. 2015 Mar 18;10(3):e0118149. doi: 10.1371/journal.pone.0118149 (PMC4364715; doi:10.1371/journal.pone.0118149)
Supplement: S2 Table — (PDF) [file pone.0118149.s003.pdf]

Table S2. Associations of 24 candidate SNPs with HIV-1 acquisition in our meta-analysis of African Americans and European Americans from the Urban Health Study. The SNPs were reported for having a suggestive association with HIV-1 acquisition, as reviewed by An and Winkler[20] or McLaren et al.[21] The SNPs are sorted by chromosomal position.

					African Americans (N=2,004)			*European Americans (N=1,132)*			*UHS meta-analysis*	
SNP	*Chr*	*Position (NCBI build 37)*	*Gene / nearby gene*	*Coded allele*	*CAF*	*P*	*OR (95% CI)*	*CAF*	*P*	*OR (95% CI)*	*P*	*Reference(s)*
rs1800872	1	206,946,407	*IL10*	T	0.40	0.84	0.99 (0.86-1.13)	0.23	0.85	1.02 (0.82-1.27)	0.96	McLaren et al.
rs3732378	3	39,307,162	*CX3CR1*	A	0.04	0.75	1.05 (0.76-1.47)	0.17	0.70	1.04 (0.83-1.32)	0.63	McLaren et al.
rs3732379	3	39,307,256	*CX3CR1*	T	0.15	0.86	1.02 (0.84-1.23)	0.28	0.85	1.02 (0.83-1.25)	0.80	McLaren et al.
rs2549782	5	96,231,000	*ERAP2*	G	0.42	0.94	1.00 (0.88-1.15)	0.47	0.25	0.90 (0.75-1.08)	0.53	McLaren et al.
rs17848424 (rs2070729)	5	131,819,921	*IRF1*	C	0.38	0.83	1.02 (0.88-1.17)	0.55	0.10	1.17 (0.97-1.41)	0.25	An & Winkler, McLaren et al.
rs17848395 (rs2070721)	5	131,825,842	*IRF1*	T	0.38	0.91	1.01 (0.87-1.16)	0.55	0.12	1.17 (0.96-1.41)	0.30	An & Winkler, McLaren et al.
rs6850	7	44,836,314	*PPIA*	G	0.45	0.13	1.11 (0.97-1.28)	0.13	0.22	1.19 (0.90-1.56)	0.051	An & Winkler, McLaren et al.
rs6996198	8	65,463,442	*BHLHE22*	T	0.21	0.33	1.09 (0.92-1.28)	0.16	0.50	0.92 (0.70-1.19)	0.71	McLaren et al.
rs1552896	9	14,841,387	*FREM1*	G	0.07	0.50	0.91 (0.69-1.19)	0.22	1.00	1.00 (0.79-1.27)	0.59	McLaren et al.
rs1801157	10	44,868,257	*CXCL12*	T	0.07	0.48	1.10 (0.85-1.43)	0.20	0.82	0.97 (0.77-1.23)	0.67	McLaren et al.
rs754618	10	44,886,206	*CXCL12*	T	0.12	0.72	0.96 (0.78-1.19)	0.31	0.59	0.94 (0.78-1.15)	0.54	McLaren et al.

rs10838525	11	5,701,001	*TRIM5*	T	0.15	0.58	1.05 (0.87-1.28)	0.36	0.60	0.95 (0.78-1.15)	0.90	An & Winkler, McLaren et al.
rs3740996	11	5,701,281	*TRIM5*	A	0.07	0.90	1.02 (0.75-1.37)	0.11	0.39	1.14 (0.85-1.52)	0.54	An & Winkler, McLaren et al.
rs16934386	11	5,706,283	*TRIM5*	G	0.08	0.19	0.84 (0.65-1.09)	0.002	0.61	0.57 (0.06-5.26)	0.18	An & Winkler
rs1946518	11	112,035,458	*IL18*	T	0.36	0.75	0.98 (0.85-1.13)	0.40	0.52	0.94 (0.78-1.13)	0.52	McLaren et al.
rs1024611	17	32,579,788	*CCL2*	G	0.19	0.47	1.06 (0.90-1.27)	0.28	0.14	0.85 (0.69-1.05)	0.75	McLaren et al.
rs1024610	17	32,580,231	*CCL2*	T	0.08	0.74	1.04 (0.81-1.34)	0.20	0.78	0.97 (0.78-1.21)	0.92	McLaren et al.
rs2857657	17	32,583,132	*CCL2*	G	0.05	0.67	1.07 (0.78-1.48)	0.20	0.99	1.00 (0.80-1.26)	0.73	McLaren et al.
rs4795895	17	32,611,446	*CCL7 / CCL11*	A	0.04	0.63	1.09 (0.78-1.52)	0.19	1.00	1.00 (0.80-1.25)	0.70	McLaren et al.
rs2280789	17	34,207,003	*CCL5*	G	0.19	0.02	0.81 (0.68-0.97)	0.13	0.08	1.28 (0.97-1.69)	0.46	An & Winkler, McLaren et al.
rs2280788	17	34,207,405	*CCL5*	C	0.01	0.90	1.06 (0.40-2.86)	0.02	0.05	1.82 (1.01-3.23)	0.20	McLaren et al.
rs2107538	17	34,207,780	*CCL5*	T	0.44	0.45	0.95 (0.83-1.09)	0.17	0.11	1.22 (0.95-1.56)	0.73	An & Winkler, McLaren et al.
rs1719134	17	34,416,946	*CCL3*	A	0.15	0.02	1.25 (1.04-1.49)	0.23	0.92	0.99 (0.80-1.22)	0.060	McLaren et al.
rs4804803	19	7,812,733	*DC-SIGN*	G	0.40	0.94	1.01 (0.88-1.15)	0.22	0.18	1.16 (0.93-1.45)	0.39	An & Winkler

CAF, coded allele frequency
