# Supplementary figures and images for: Novel Genetic Locus Implicated for HIV-1 Acquisition with Putative Regulatory Links to HIV Replication and Infectivity: A Genome-Wide Association Study
Source: PLoS One. 2015 Mar 18;10(3):e0118149. doi: 10.1371/journal.pone.0118149 (PMC4364715; doi:10.1371/journal.pone.0118149)

**S1 Figure.** Best fitting latent class model of HIV risk behavior among IDUs

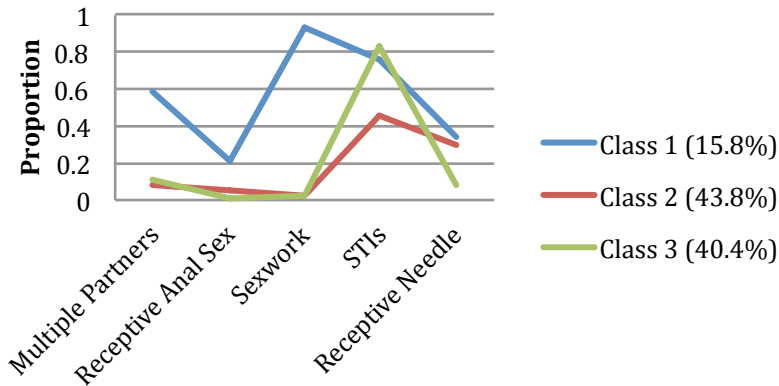

Supplement: S1 Fig — (PDF) [file pone.0118149.s006.pdf]
