# Supplementary material for: Novel Genetic Locus Implicated for HIV-1 Acquisition with Putative Regulatory Links to HIV Replication and Infectivity: A Genome-Wide Association Study
Source: PLoS One. 2015 Mar 18;10(3):e0118149. doi: 10.1371/journal.pone.0118149 (PMC4364715; doi:10.1371/journal.pone.0118149)

Figure S2. STRUCTURE triangle plots showing estimated ancestral proportions of African American and European American participants with reference to HapMap populations. Ancestral proportion estimates in (A) African Americans and (B) European Americans were based on 10,000 randomly selected HapMap phase III SNPs. The triangle's vertices represent West Africans, European Americans, and East Asians, and the triangle's edges indicate the ancestral proportions. These estimates were used as a quality control procedure to corroborate self-reported ancestry. Ancestry principal components were subsequently calculated for inclusion as covariates in the GWAS analyses to reduce any potential bias from population stratification.

(A)

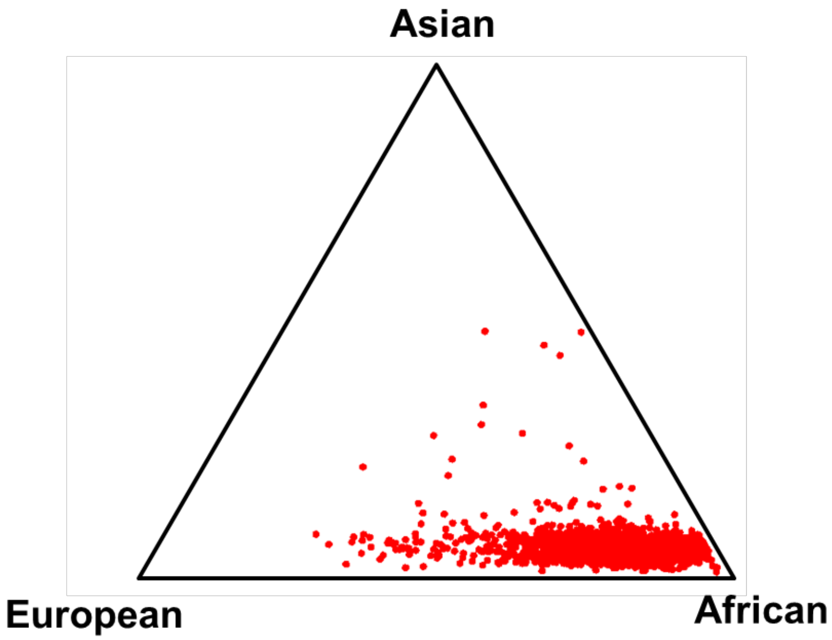

(B)

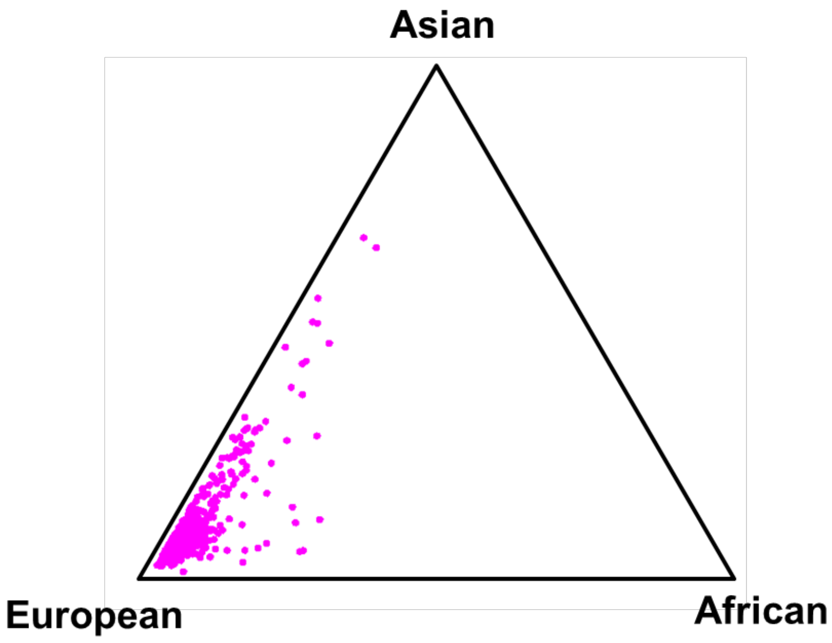

Supplement: S2 Fig — (PDF) [file pone.0118149.s007.pdf]
