# Supplementary material for: Novel Genetic Locus Implicated for HIV-1 Acquisition with Putative Regulatory Links to HIV Replication and Infectivity: A Genome-Wide Association Study
Source: PLoS One. 2015 Mar 18;10(3):e0118149. doi: 10.1371/journal.pone.0118149 (PMC4364715; doi:10.1371/journal.pone.0118149)

Figure S3. Genome-wide association study of HIV-1 acquisition in 2,004 African Americans from the Urban Health Study. (A) The quantile-quantile plot shows the association results of approximately 8 million tested SNPs and indels. The observed distribution of *P* values vs. the expected distribution of *P* values (black dots) is plotted on a logarithmic scale along with the identity line (red), which represents identical observed and expected distributions. The corresponding genomic inflation factor ($\lambda_{gc} = 1.018$) is shown. (B) The $-\log_{10}$ (*P* value) are plotted by chromosomal position of SNPs (shown as circles) and indels (shown as triangles). The solid line indicates the threshold for declaring genome-wide statistical significance ($P<5x10^{-8}$).

(A)

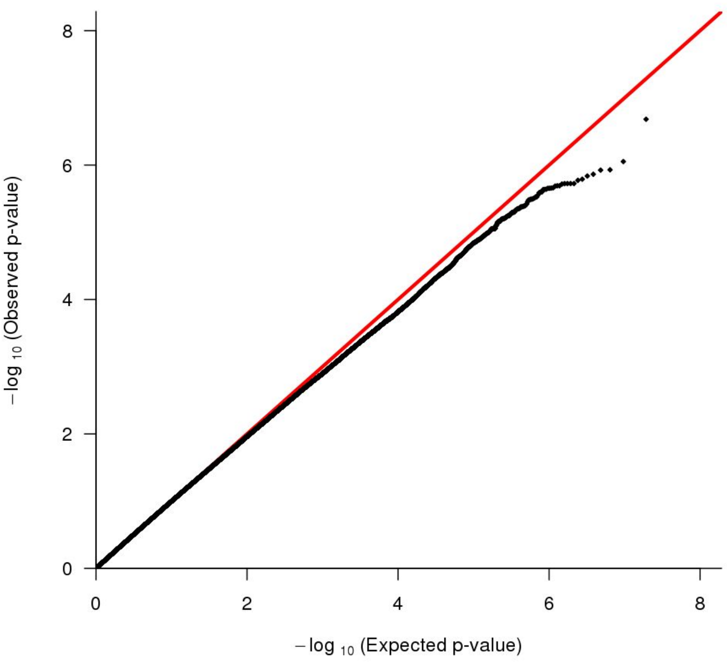

(B)

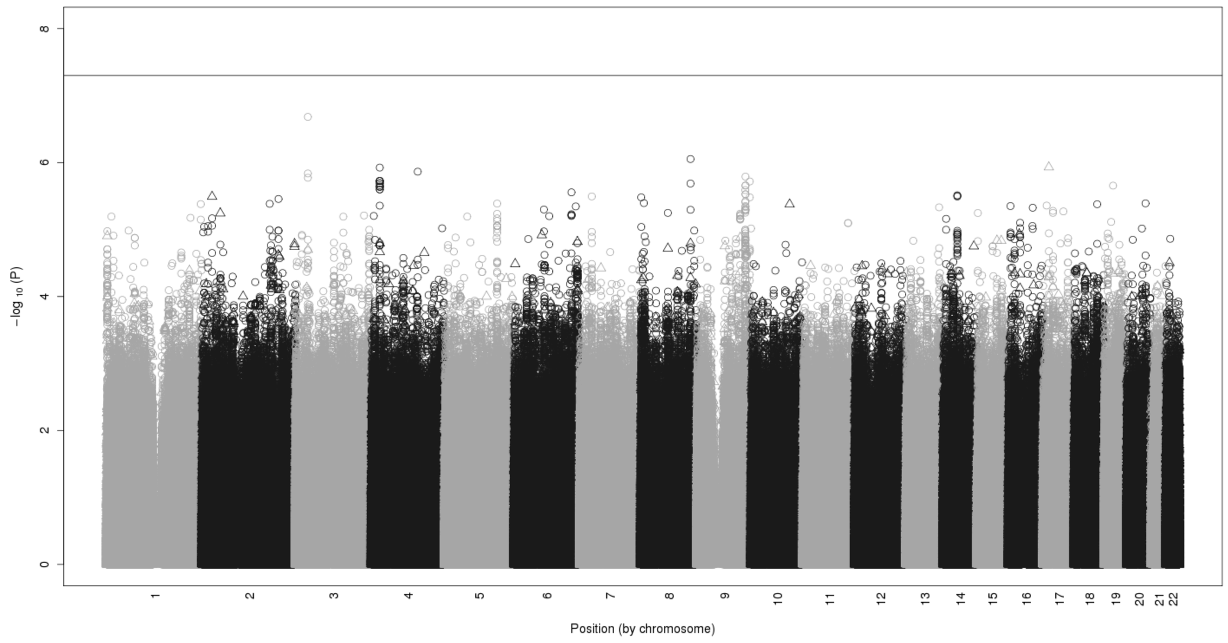

Supplement: S3 Fig — (PDF) [file pone.0118149.s008.pdf]
