# Supplementary material for: Novel Genetic Locus Implicated for HIV-1 Acquisition with Putative Regulatory Links to HIV Replication and Infectivity: A Genome-Wide Association Study
Source: PLoS One. 2015 Mar 18;10(3):e0118149. doi: 10.1371/journal.pone.0118149 (PMC4364715; doi:10.1371/journal.pone.0118149)

Figure S5. Quantile-quantile plot showing the meta-analysis results of approximately 8 million SNPs and indels tested for association with HIV-1 acquisition in 2,004 African Americans and 1,132 European Americans from the Urban Health Study. The observed distribution of meta-analysis *P* values vs. the expected distribution of *P* values (black dots) is plotted on a logarithmic scale along with the identity line (red), which represents identical observed and expected distributions. The corresponding genomic inflation factor (λ_{gc}) is shown.

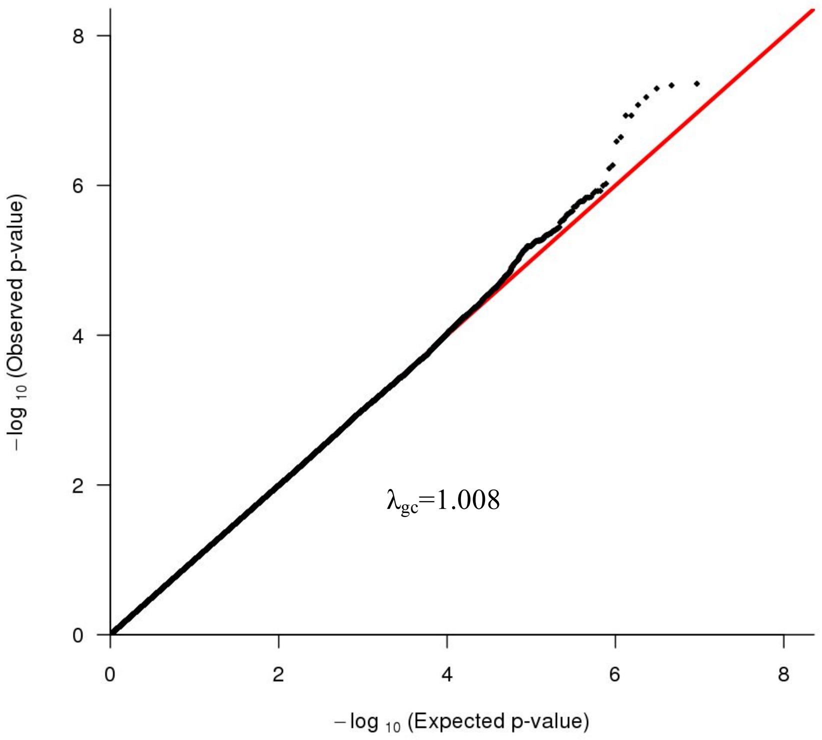

Supplement: S5 Fig — (PDF) [file pone.0118149.s010.pdf]
