# Supplementary material for: Novel Genetic Locus Implicated for HIV-1 Acquisition with Putative Regulatory Links to HIV Replication and Infectivity: A Genome-Wide Association Study
Source: PLoS One. 2015 Mar 18;10(3):e0118149. doi: 10.1371/journal.pone.0118149 (PMC4364715; doi:10.1371/journal.pone.0118149)
Supplement: S6 Fig — (PDF) [file pone.0118149.s011.pdf]

Figure S6. Regional association results from the GWAS meta-analysis in the Urban Health Study and their linkage disequilibrium patterns with reference to the 1000 Genomes AFR panel. The r^2 correlations between the SNP with the lowest meta-analysis *P* value (shown in purple) and its surrounding SNPs selected for replication testing (meta-analysis $P<1x10^{-3}$) are shown for each of the following 8 regions: (A) *SIGLEC17P* / *CD33*, (B) *SPOCK1*, (C) *IRF2BP2* / *TOMM20*, (D) *KDM4C*, (E) *IFFO2* / *UBR4*, (F) *HLA-DQA1* / *HLA-DQB1*, (G) *KIAA1671*, and (H) *PAX5* / *ZCCHC7*. The blue horizontal dotted line indicates the meta-analysis $P<1x10^{-3}$ threshold for selecting SNPs for replication testing. Plots are oriented to capture the SNPs selected for replication within each 3MB region examined rather than being centered on the highest association peak. SNPs are intergenic or intronic (solid circles), coding (nonsynonymous SNPs shown as inverted triangles and untranslated or synonymous SNPs shown as squares), located in a conserved transcription factor binding site (stars), or highly conserved in placental mammals (known as MCS44 Placental, squares with diagonal lines). Gene locations and their orientations are shown along with recombination peaks in light blue. SNPs with missing r^2 values are shown in grey.

(A)

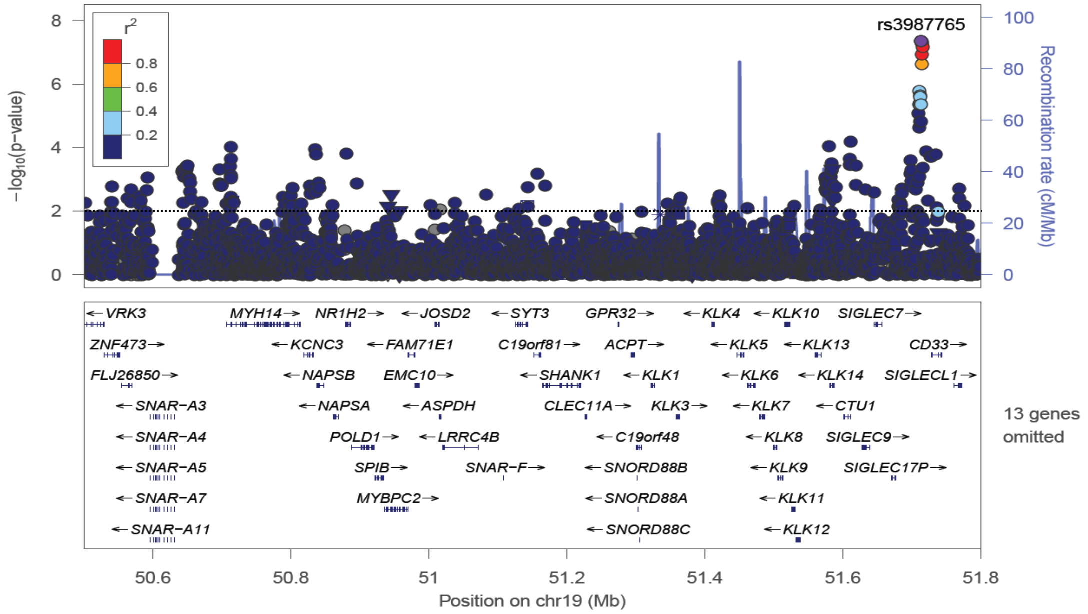

(B)

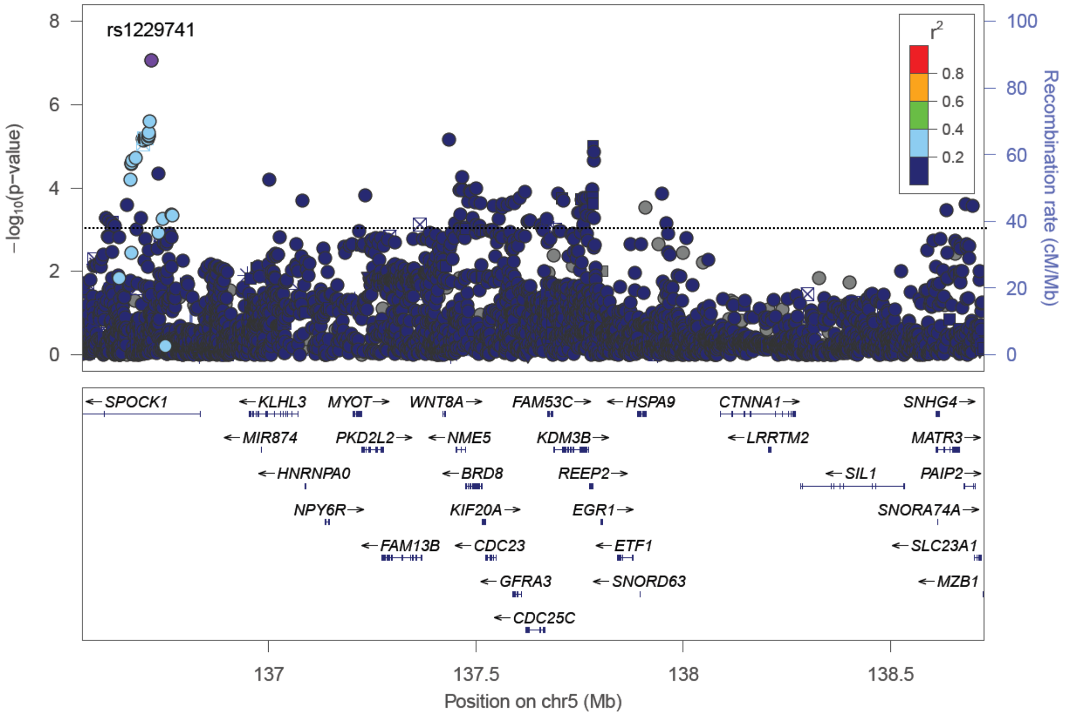

(C)

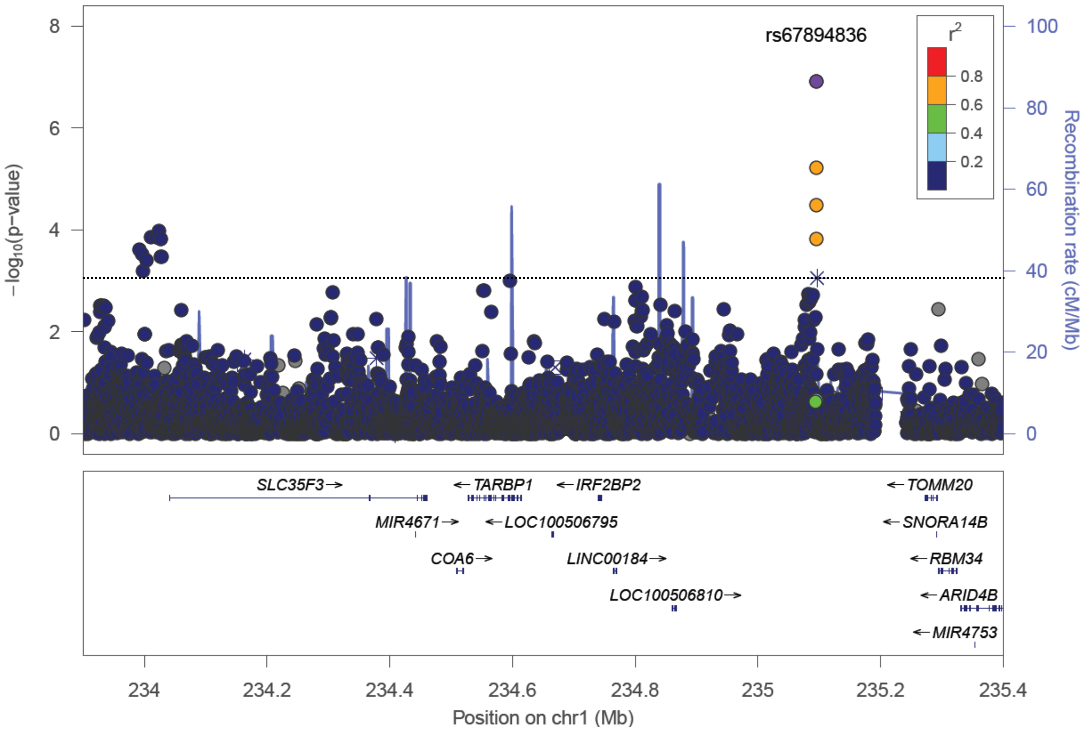
rs67894836
r2
0.8
0.6
0.4
0.2
−log10(p−value)
Recombination rate (cM/Mb)
SLC35F3→
←TARBP1
←IRF2BP2
←TOMM20
MIR4671→
←LOC100506795
←SNORA14B
COA6→
LINC00184→
←RBM34
LOC100506810→
←ARID4B
←MIR4753
Position on chr1 (Mb)

(D)

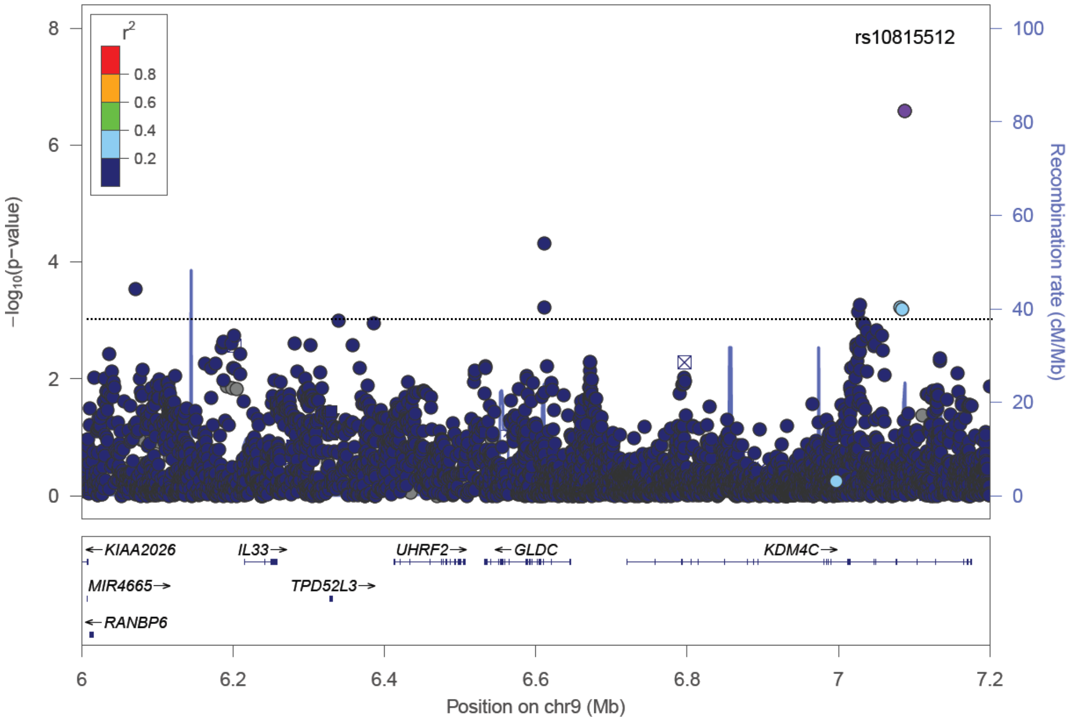
r2
0.8
0.6
0.4
0.2
rs10815512
−log10(p−value)
Recombination rate (cM/Mb)
←KIAA2026
IL33→
UHRF2→
←GLDC
KDM4C→
MIR4665→
TPD52L3→
←RANBP6
Position on chr9 (Mb)

(E)

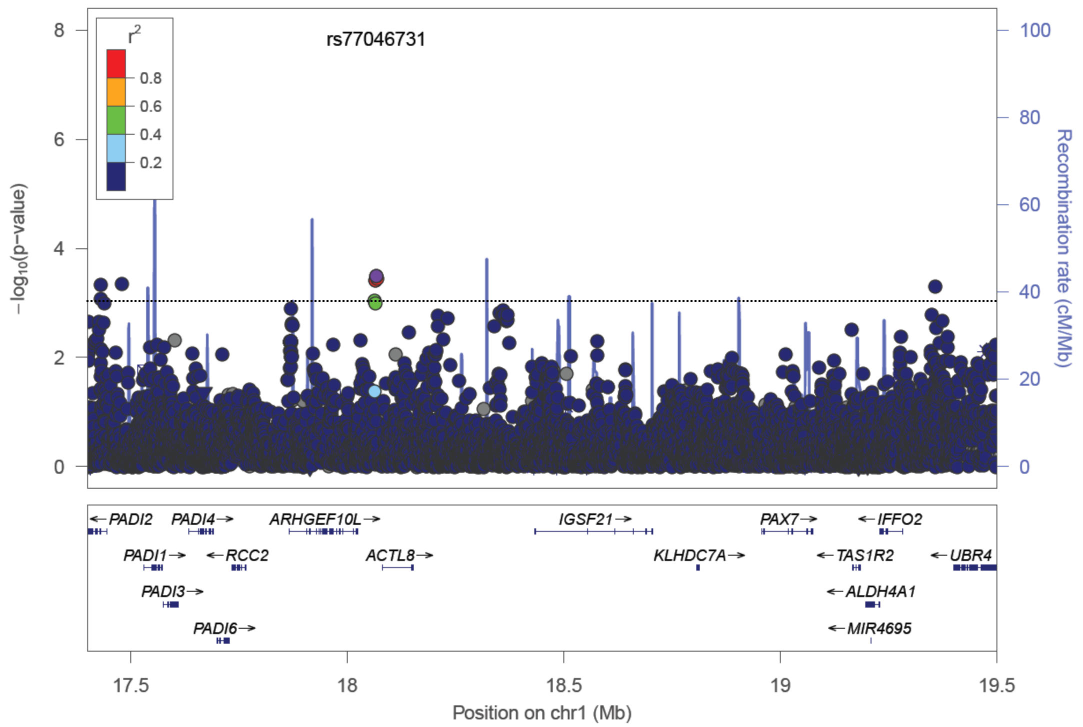

The top associated indel (chr1:19357344:D, not shown) is intergenic between *IFFO2* and *UBR4*.

(F)

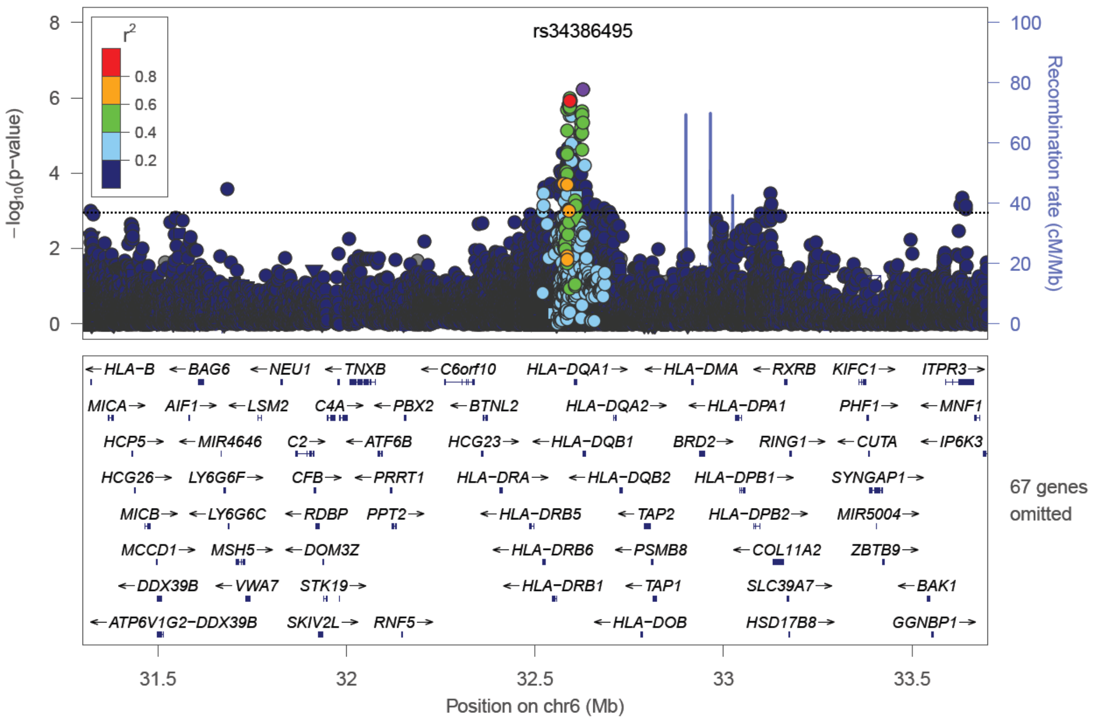

(G)

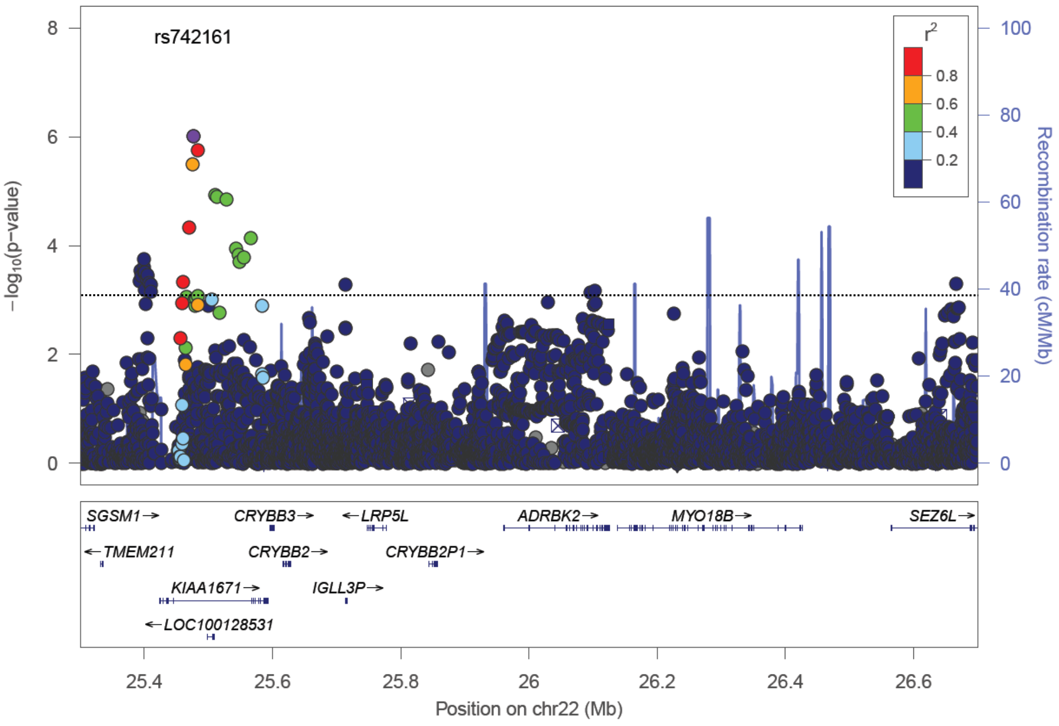
rs742161
r²
0.8
0.6
0.4
0.2
−log₁₀(p−value)
Recombination rate (cM/Mb)
SGSM1→
CRYBB3→
←LRP5L
ADRBK2→
MYO18B→
SEZ6L→
←TMEM211
CRYBB2→
CRYBB2P1→
KIAA1671→
IGLL3P→
←LOC100128531
25.4
25.6
25.8
26
26.2
26.4
26.6
Position on chr22 (Mb)

(H)

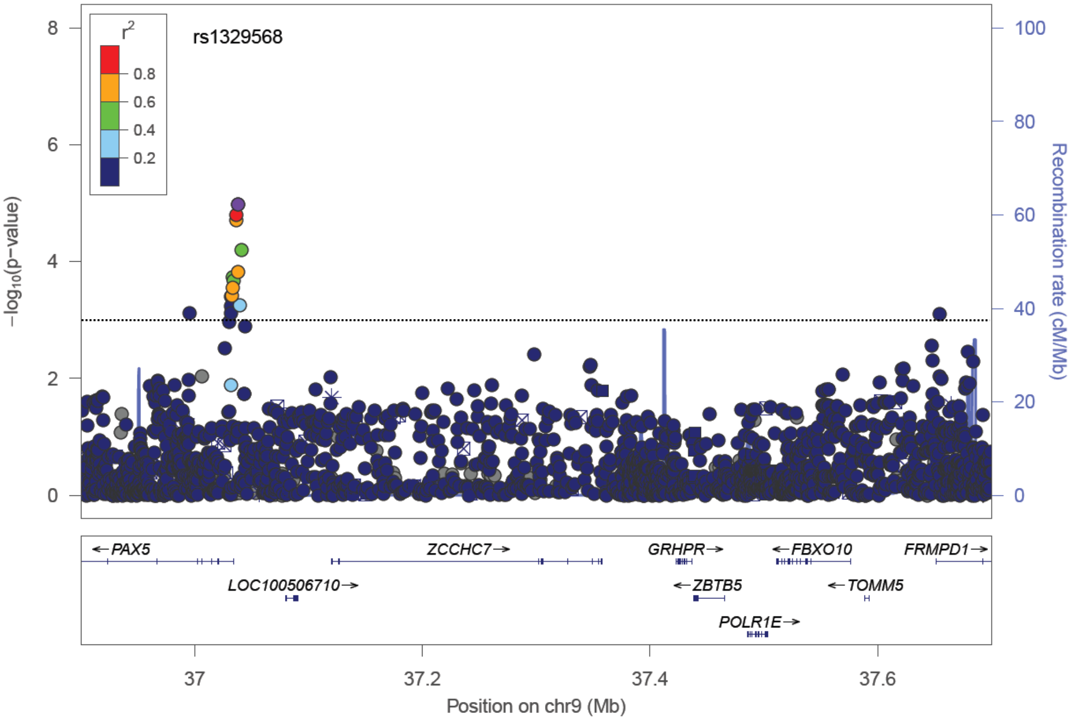
rs1329568
r²
0.8
0.6
0.4
0.2
−log₁₀(p−value)
Recombination rate (cM/Mb)
←PAX5
ZCCHC7→
GRHPR→
←FBXO10
FRMPD1→
LOC100506710→
←ZBTB5
←TOMM5
POLR1E→
37
37.2
37.4
37.6
Position on chr9 (Mb)
