# Supplementary material for: Novel Genetic Locus Implicated for HIV-1 Acquisition with Putative Regulatory Links to HIV Replication and Infectivity: A Genome-Wide Association Study
Source: PLoS One. 2015 Mar 18;10(3):e0118149. doi: 10.1371/journal.pone.0118149 (PMC4364715; doi:10.1371/journal.pone.0118149)

Figure S10. Location of gene expression probes tested for replication of RNAseq association between rs4878712 and *FBXO10*. The strongest RNAseq signal (r=-0.49, P=6.9 x 10^{-5}) was significantly associated with exon 11 in Montgomery et al. 2010 data (blue). The microarray probe associated with rs4878712 (β = -0.028, *P*=0.0176; MuTHER resource [17]) is shown in the green, and the other microarray probe not associated with rs4878712 (MuTHER resource P=0.962; Stranger et al. 2012[18]; *P*= 0.567) is shown in red.

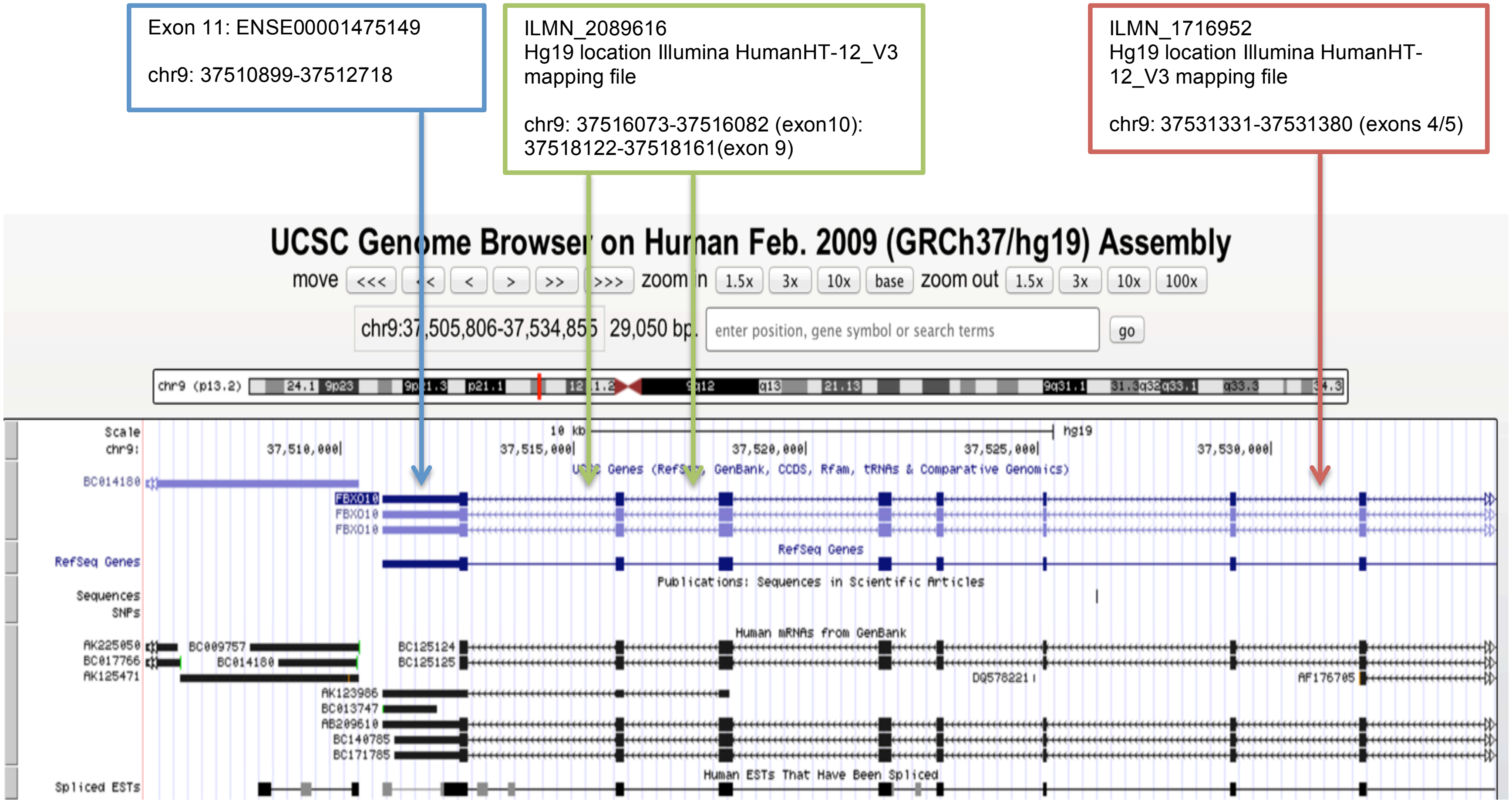

Supplement: S10 Fig — (PDF) [file pone.0118149.s015.pdf]
