# Supplementary material for: Novel Genetic Locus Implicated for HIV-1 Acquisition with Putative Regulatory Links to HIV Replication and Infectivity: A Genome-Wide Association Study
Source: PLoS One. 2015 Mar 18;10(3):e0118149. doi: 10.1371/journal.pone.0118149 (PMC4364715; doi:10.1371/journal.pone.0118149)

Figure S9. Linkage disequilibrium patterns in the 1000 Genomes EUR reference panel for the GWAS-implicated region spanning from *PAX5* to *FRMPD1* on chromosome 9. The patterns, according to (A) D' and (B) r^2 values, are shown for the 15 GWAS-implicated SNPs tested for replication on chromosome 9. Darker shading from pink to red indicates higher D' values, and darker shading from grey to black indicates higher r^2 values. Purple coloring indicates high D' values but lower levels of statistical significance.

(A)

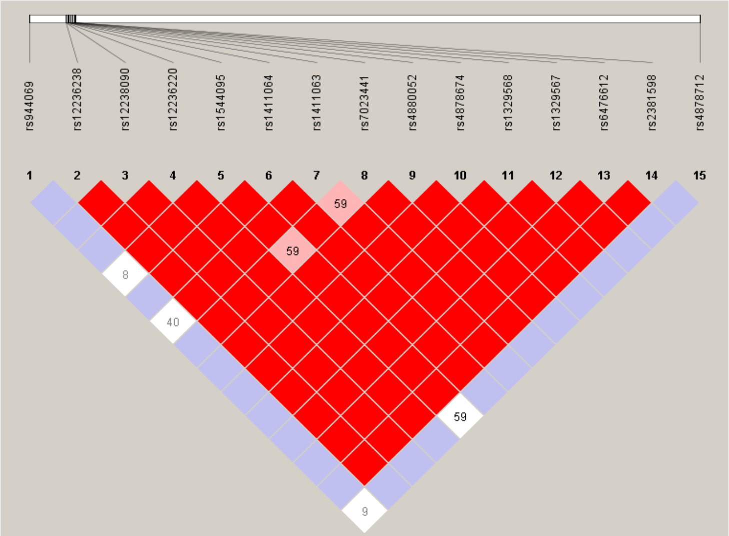

(B)

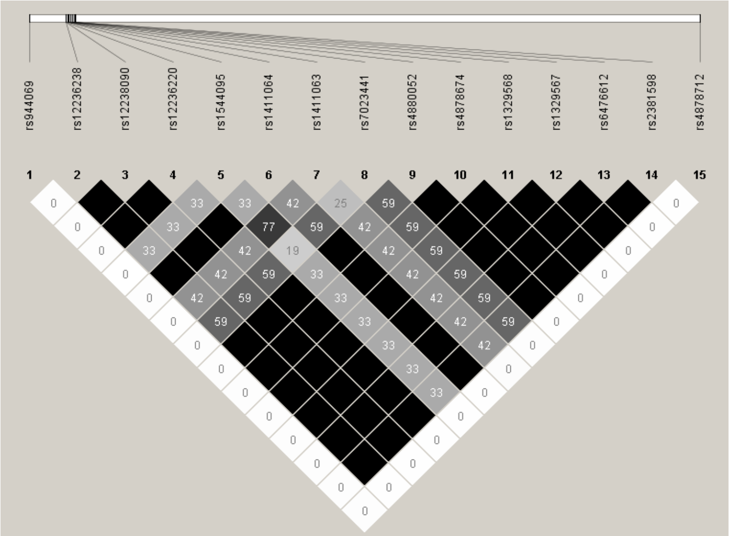

Supplement: S9 Fig — (PDF) [file pone.0118149.s014.pdf]
